# A Potent Micron Neoantigen Tumor Vaccine GP‐Neoantigen Induces Robust Antitumor Activity in Multiple Tumor Models

**DOI:** 10.1002/advs.202201496

**Published:** 2022-06-16

**Authors:** Zhe Jing, Shuqing Wang, Keyuan Xu, Qian Tang, Wenjing Li, Wei Zheng, Haobo Shi, Kailing Su, Yanting Liu, Zhangyong Hong

**Affiliations:** ^1^ State Key Laboratory of Medicinal Chemical Biology Tianjin Key Laboratory of Protein Sciences College of Life Sciences Nankai University Tianjin 300071 P. R. China; ^2^ Department of Oncology The First Affiliated Hospital of Xinxiang Medical University Weihui Henan Province 453100 P. R. China

**Keywords:** 3‐glucan particles, neoantigen, therapeutic vaccine, tumor immunotherapy, *β*‐1

## Abstract

Therapeutic tumor neoantigen vaccines have been widely studied given their good safety profile and ability to avoid central thymic tolerance. However, targeting antigen‐presenting cells (APCs) and inducing robust neoantigen‐specific cellular immunity remain challenges. Here, a safe and broad‐spectrum neoantigen vaccine delivery system is proposed (GP‐Neoantigen) based on *β*‐1,3‐glucan particles (GPs) derived from Saccharomyces cerevisiae and coupling peptide antigens with GPs through convenient click chemistry. The prepared system has a highly uniform particle size and high APC targeting specificity. In mice, the vaccine system induced a robust specific CD8^+^ T cell immune response and humoral immune response against various conjugated peptide antigens and showed strong tumor growth inhibitory activity in EG7·OVA lymphoma, B16F10 melanoma, 4T1 breast cancer, and CT26 colon cancer models. The combination of the toll‐like receptors (TLRs) agonist PolyI:C and CpG 2395 further enhanced the antitumor response of the particle system, achieving complete tumor clearance in multiple mouse models and inducing long‐term rejection of reinoculated tumors. These results provide the broad possibility for its further clinical promotion and personalized vaccine treatment.

## Graphical abstract

A broad‐spectrum neoantigen vaccine delivery system (GP‐Neoantigen) based on *β*‐1,3‐glucan particles (GPs) derived from Saccharomyces cerevisiae induces a robust cellular and humoral immune response against conjugated peptide antigens and strongly inhibits tumor growth, with which complete tumor clearance and long‐term rejection of reinoculated tumors can be achieved in multiple mouse tumor models.

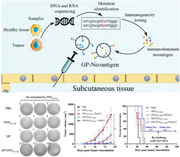

## 1 Introduction

Tumor‐specific neoantigens, generated from nonsynonymous point mutations in tumor genes, are regarded as ideal antigens for inducing tumor immune rejection.^[^ ^1^ ^]^ These neoantigens are not affected by central immune tolerance and thus can induce a strong immune response and avoid the risk of autoimmune diseases.^[^ ^2^ ^]^ Personalized tumor vaccines based on neoantigens have become an important branch of cancer immunotherapy, and a large number of clinical trials are being performed.^[^ ^3^ ^]^ However, how to induce the body to generate a high‐quantity neoantigen‐specific T cell immune response remains a key technical challenge in this field.^[^ ^4^ ^]^ At present, the main immune strategies occur through the long peptides of neoantigens combined with PolyI:C adjuvant^[^ ^5^ ^]^ or mRNA vaccine technology,^[^ ^6^ ^]^ but their efficiency remains limited. Some particulate vaccine systems based on synthetic polymers, liposomes, and membrane vesicles have been developed to significantly improve the efficiency of T cellular immune responses and therapeutic results in animal models.^[^ ^7^ ^]^ However, the vaccine particles prepared by these systems are usually uneven in size and surface structure or inconsistent among batches. The size and shape of vaccine particles have a significant impact on their immune performance; thus, heterogeneity hinders activity evaluation and clinical application.^[^ ^8^ ^]^

## 2 Results and Discussion

**Figure 1:**
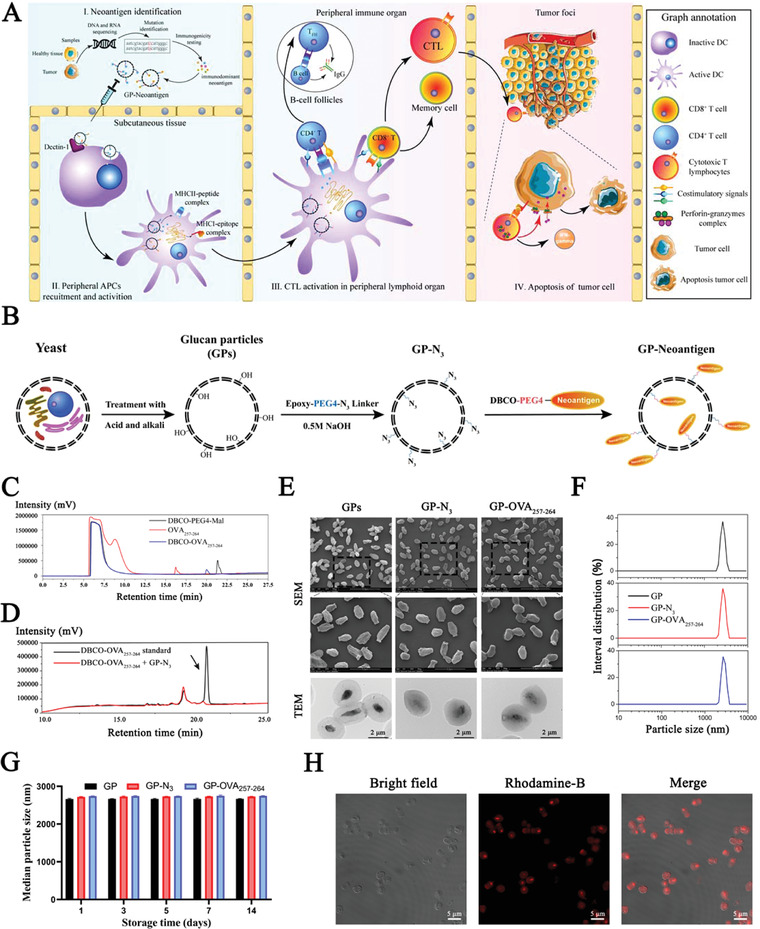
Characterization of the GP‐Neoantigen antigen loading system. A) Schematic illustration of the GP‐Neoantigen vaccine to activate antitumor immune responses and induce tumor apoptosis. B) Schematic graph of the preparation of the GP‐Neoantigen antigen loading system. C) The reaction efficiency of cysteine‐OVA_257‐264_ and DBCO‐PEG4‐maleimide is monitored by HPLC. Red lines indicate different standard cysteine‐OVA_257‐264_, black lines indicate DBCO‐PEG4‐maleimide, and blue lines indicate the high‐purity product. D) The conjugation efficiency of DBCO‐OVA_257‐264_ to GP‐N_3_ is monitored by HPLC. The black line indicates DBCO‐OVA_257‐264_. Arrows indicate actual DBCO‐OVA_257‐264_, whereas another minor peak is slightly excessive unreacted cysteine‐peptide. The red line indicates supernatant after the conjugation reaction. E) Scanning electron microscopy and transmission electron microscopy images of GP, GP‐N_3_, and GP‐OVA_257‐264_. F,G) Particle size of GP, GP‐N_3_, and GP‐OVA_257‐264_ immediately after preparation (F) or at different times after storage at 37 °C (G) analyzed by dynamic light scattering. H) CLSM images of GP particles with RhoB‐labeled OVA_257‐264_ peptide coupling on the surface of GPs.

Here, we propose a safe and broad‐spectrum peptide neoantigen vaccine system (GP‐Neoantigen) based on *β*‐1,3‐glucan particles (GPs) derived from natural edible Saccharomyces cerevisiae (**Figure** **1**A). GPs are hollow yeast shell particles obtained by removing nucleic acids and proteins inside and outside the yeast cells through simple acid and alkali treatment.^[^ ^9^ ^]^ The ingredient *β*‐glucan is a highly safe food additive approved by the US Food and Drug Administration as a generally recognized as safe (GRAS) product^[^ ^10^ ^]^ and has immune‐activating properties.^[^ ^11^ ^]^ GPs naturally have a uniform size in structure (approximately 2–4 µm). In addition, the process of removing nucleic acids and proteins does not change their size and shape at all, so the particles prepared from batch to batch have a high degree of consistency.^[^ ^9^ ^]^ Moreover, due to the ideal particle size and the use of *β*‐1,3‐D‐glucan as the ligand of the dectin‐1 receptor,^[^ ^12^ ^]^ the particle system has high target specificity for uptake by antigen‐presenting cells (APCs), such as dendritic cells (DCs) and macrophages,^[^ ^13^ ^]^ whereas it will not be taken up by other types of cells. Thus, the particles can avoid the reduction of immune activation due to nonspecific uptake common in conventional vaccine delivery systems. In addition, the *β*‐1,3‐D‐glucan backbone is also a powerful pattern recognition ligand^[^ ^14^ ^]^ that can efficiently activate the T_H_1‐biased cellular immune response.^[^ ^15^ ^]^ These factors all indicate that GPs may represent a very promising vaccine delivery system for peptide neoantigens.

We first selected the major histocompatibility complex I (MHC‐I)‐restricted peptide OVA_257‐264_ (SIINFEKL, from OVA protein) as the model antigen^[^ ^16^ ^]^ to test and optimize the immune activation function of the GPs as a neoantigen delivery system. Here, we loaded OVA_257‐264_ onto GPs by covalent chemical coupling (Figure 1B). We tried a variety of strategies. Finally, we first obtained GP‐N_3_ through the epoxy ring‐opening reaction of the alcoholic hydroxyl group on the GPs and the epoxy group on the epoxy‐PEG_4_‐N_3_ linker under alkaline conditions and then coupled it with DBCO‐modified OVA_257‐264_ (DBCO‐OVA_257‐264_) through the click reaction to obtain GP‐OVA_257‐264_ vaccine particles. High‐performance liquid chromatography (HPLC) showed that the preparation of DBCO‐OVA_257‐264_ and the coupling of DBCO‐OVA_257‐264_/GP‐N_3_ were very efficient (Figure 1C,D and Table S1, Supporting Information). Scanning/transmission electron microscopy and dynamic light scattering showed that the size of these particles was 2–4 µm. In addition, modification with azido or OVA_257‐264_ did not change the particle size, but the surface of modified GPs seemed to become smoother and less transparent (Figure 1E,F; Table S2, Figure S1, Supporting Information). The absolute value of zeta potential of GP‐N_3_ and GP‐OVA_257‐264_ is slightly lower than that of the GPs, which may be related to surface modification of azide groups and OVA_257‐264_ peptides (Figure S2, Supporting Information). The prepared GP‐OVA_257‐264_ particles were highly stable, remaining unchanged in size after storage in saline at 37 °C for 14 days (Figure 1G). Confocal laser scanning microscopy (CLSM) imaging revealed that in GP‐OVA_257‐264_‐RhoB particles where GPs were conjugated with rhodamine B (RhoB)‐labeled OVA_257‐264_ (OVA_257‐264_‐RhoB), RhoB was colocalized with the outer wall of GPs, again showing that the peptide was successfully coupled to the GP shell.

**Figure 2:**
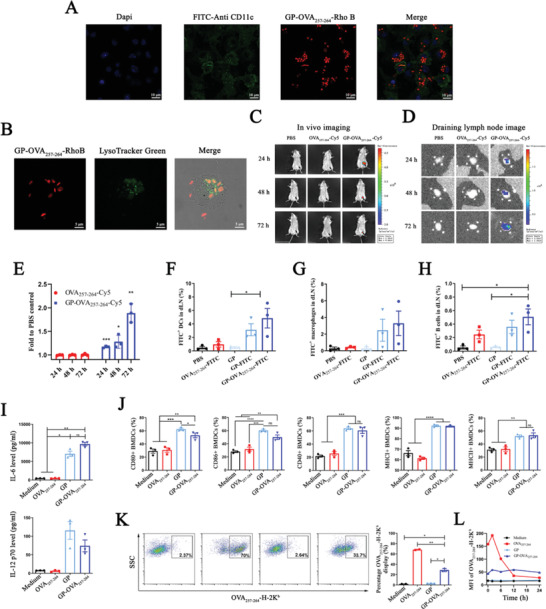
Uptake of GP‐OVA_257‐264_ particles by APCs induced activation and cross‐presentation. A) CLSM images of GP‐OVA_257‐264_ uptake by BMDCs. BMDCs are incubated with GP‐OVA_257‐264_‐RhoB for 24 h, and the nucleus and cell membrane are separately labeled with DAPI‐ and FITC‐conjugated CD11c antibodies. B) CLSM images of lysosomal localization of GP‐OVA_257‐264_ particles in BMDCs. BMDCs are incubated with GP‐OVA_257‐264_‐RhoB for 24 h, and the lysosomes are labeled with Lysotracker Green. C–E) Live imaging of the location and migration of GP‐OVA_257‐264_ in vivo. BALB/c mice are subcutaneously inoculated with GP‐OVA_257‐264_‐Cy5 or controls, and the fluorescence images of inoculated mice (C), draining lymph nodes (D), and relative fluorescence intensity of Cy5 in draining lymph nodes compared to PBS inoculated mice (E) at 24, 48, and 72 h after immunization are shown. The black arrow in (C) represents the injection site. F–H) FACS analysis of in vivo uptake by APCs. C57BL/6 mice are subcutaneously inoculated with FITC‐labeled OVA_257‐264_, GP, or GP‐OVA_257‐264_‐FITC in the inguinal area, and the draining lymph nodes are ingested 24 h later. The uptake ratios of DCs (F), macrophages (G), and B cells (H) are analyzed by FACS. PBS and FITC‐unlabeled GPs are used as negative controls. I, J) Activation of BMDCs by GP‐OVA_257‐264_. BMDCs are incubated with GP‐OVA_257‐264_ and controls. IL‐6 and IL‐12 p70 release is detected by ELISA (I), and the expression of the activation markers CD80, CD86, CD40, MHC‐I, and MHC‐II is detected by FACS (J). K,L) FACS analysis of OVA_257‐264_ peptide cross‐presentation efficiency in BMDCs. Representative flow cytometry graph and quantified frequency of OVA_257‐264_‐H‐2K^b^ displayed BMDCs at 2 h (K). A dynamic graph of the cross‐presentation efficiency of BMDCs (L) is shown. Data represent the mean ± SEM. Statistical significance is calculated by one‐way ANOVA with Tukey's significant difference multiple comparisons. * *p* < 0.05, ** *p* < 0.01, *** *p* < 0.001, **** *p* < 0.0001.

Then, we examined the specific uptake and intracellular degradation of GP‐OVA_257‐264_ by APCs. Here, we cocultured bone marrow‐derived DCs (BMDCs) or bone marrow‐derived macrophages (BMDMs) with GP‐OVA_257‐264_‐RhoB. CLSM imaging showed that the particles (red) were efficiently taken up by BMDCs and BMDMs (**Figure** **2**A; Figure S3, Supporting information), whereas free OVA_257‐264_‐RhoB was not (Figure S4, Supporting Information). It is worth mentioning that these particles were not taken up by nonphagocytic cells, such as 3T3, 293T, and LO2 cells, and rarely by neutrophils (Figure S5, Supporting Information), demonstrating that these particles have high targeting specificity for APC phagocytosis. GP‐OVA_257‐264_‐RhoB taken up by cells colocalized with lysosomes (Figure 2B), which could facilitate the degradation of GPs to release the antigens. Further CLSM revealed that BMDCs and BMDMs displayed rapid fluorescence decay 48 h after phagocytosis (Figures S6 and S7, Supporting Information), suggesting that GP‐OVA_257‐264_ particles were degraded by APCs within one to two days. Subsequently, we evaluated the absorption and distribution of GP‐OVA_257‐264_ in mice. In vivo imaging of the ​​C57BL/6 mice inoculated with Cy5‐labeled GP‐OVA_257‐264_‐Cy5 and OVA_257‐264_‐Cy5 showed that the fluorescence signal of GP‐OVA_257‐264_‐Cy5‐inoculated mice remained after 72 h at the injection site and draining lymph nodes (Figure 2C–E), and no fluorescence signal was observed in other organs (Figure S8, Supporting Information). The signals of the OVA_257‐264_‐Cy5 injection group completely disappeared within 24 h. We subcutaneously injected FITC‐labeled GP‐OVA_257‐264_‐FITC particles and OVA_257‐264_‐FITC into the mice, and the DCs, macrophages or B cells in the draining lymph nodes highly and efficiently took up the particles but not free peptides (Figure 2F–H; Figure S9, Supporting Information). These results further confirmed that GP‐OVA_257‐264_ was effectively taken up by APCs and significantly prolonged the residence time of the antigen in vivo.

Next, we explored the immune activation capability of GP‐OVA_257‐264_. We cocultured BMDCs, BMDMs, or RAW264.7 cells with GP‐OVA_257‐264_ and controls. Enzyme‐linked immunosorbent assay (ELISA) analysis revealed that the expression of the proinflammatory cytokines IL‐6 and IL‐12 in the coculture supernatant was significantly upregulated (Figure 2I). FACS showed that the expression of costimulatory signals, such as CD80, CD86, CD40, MHC‐I, and MHC‐II, on the surface of these cells was also significantly upregulated (Figure 2J; Figures S10, S11, and S12, Supporting Information). *β*‐Glucan on the surface of GPs is a natural pattern recognition molecule that can strongly promote the immune activation of APCs, and coupling with peptides does not change this characteristic. Subsequently, we examined the capability of antigen cross‐presentation by APCs induced by GP‐OVA_257‐264_. BMDCs were cocultured with GP‐OVA_257‐264_ and controls and then stained with APC‐conjugated anti‐H‐2Kb bound to OVA_257‐264_ monoclonal antibody. FACS showed that incubation with GP‐OVA_257‐264_ for 42 h could induce greater than 30% of BMDCs that presented OVA_257‐264_ (Figure 2K). Incubation with free OVA_257‐264_ could also induce antigen presentation by BMDCs due to its direct binding to MHC‐I. However, long‐term monitoring showed that GP‐OVA_257‐264_ induced sustained and stable antigen presentation for up to 24 h, whereas the presentation induced by free OVA_257‐264_ decayed rapidly after reaching a peak at approximately 2 h (Figure 2L; Figure S13, Supporting Information). Furthermore, GP‐OVA_257‐264_ and free GPs showed minimal cytotoxicity to BMDCs at a concentration of 1.0 mg mL^−1^ (Figure S14, Supporting Information).

**Figure 3:**
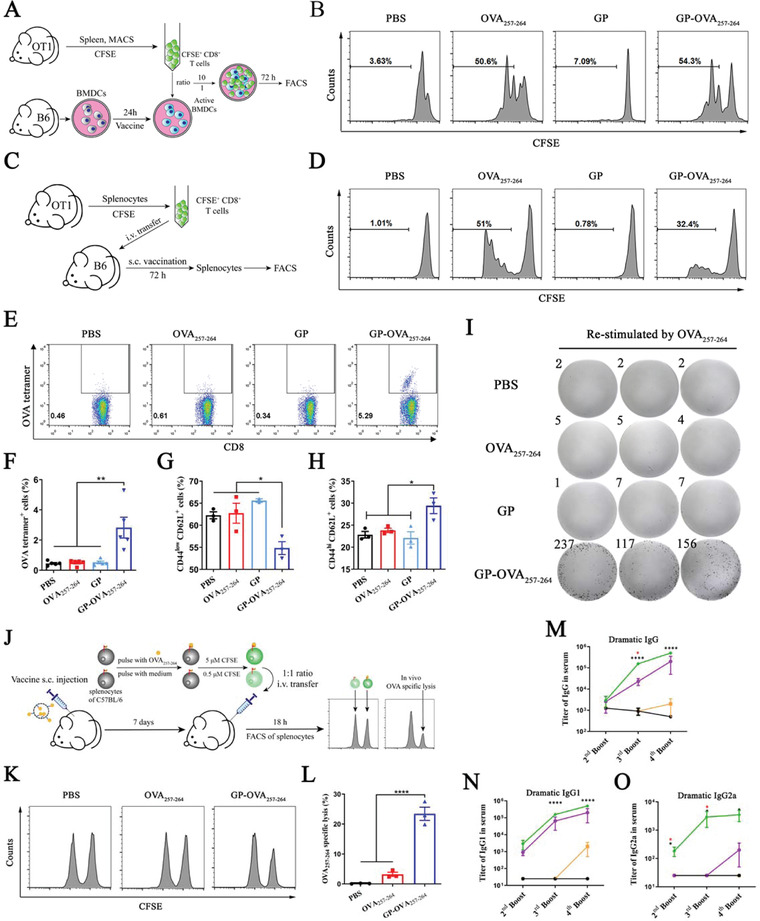
Induced cellular and humoral immune responses by OVA peptide‐conjugated GP particles. A,B) In vitro OT‐1 TCR transgenic CD8^+^ T cell proliferation stimulated with GP‐OVA_257‐264_ particles and controls. A schematic graph of the in vitro OT‐1 proliferation assay (A) and a histogram graph of FACS (B) are displayed. C,D) In vivo OT‐1 TCR transgenic CD8^+^ T cell proliferation stimulated with GP‐OVA_257‐264_ particles and controls. A schematic graph of the in vivo OT‐1 proliferation assay (C) and a histogram graph of FACS (D) are displayed. E–I) Proliferation and activation of OVA_257‐264_‐specific CD8^+^ T cells. C57BL/6 mice are inguinal inoculated twice with GP‐OVA_257‐264_ and controls every 14 days. Then, the representative flow cytometry graph (E), quantified frequency (F) of OVA_257‐264_‐specific CD8^+^ T cells, and the frequency of naïve (G) or central memory CD8^+^ T cells (H) in splenocytes are measured by FACS 7 days after the second immunization. The splenocytes of inoculated mice are restimulated with OVA_257‐264_ ex vivo, and IFN‐*γ* ^+^ CD8^+^ T cell proliferation is detected by ELISpot assay (I). J–L) OVA‐specific CD8^+^ T cell lysis assay in vivo. Schematic graph of the in vivo CD8^+^ T cell lysis assay (J), histogram images (K), and cell lysis ratio (L) are shown. M–O) ELISA of OVA‐specific antibody titers. C57BL/6 mice are inguinally inoculated with GP‐OVA_323‐339_ and controls, and peripheral blood is obtained on Day 7 after the 2nd, 3rd, and 4th immunizations for ELISA analysis. Dynamic antibody titers of OVA‐specific IgG (M), IgG1 (N), and IgG2a (O) are shown. Data represent the mean ± SEM. Statistical significance is calculated by one‐way ANOVA with Tukey's significant difference multiple comparisons. * *p* < 0.05, ** *p* < 0.01, **** *p* < 0.0001. Black * in (M–O) versus OVA_323‐339_, red * in (M,O) versus OVA.

Next, we explored the ability of GP‐OVA_257‐264_ particles to induce an antigen‐specific CD8^+^ T cell immune response. We first examined the effects of CD8^+^ T cells (OT‐1 cells) from OT‐1 TCR transgenic mice through in vitro and in vivo expansion experiments.^[^ ^17^ ^]^ For the in vitro experiment (**Figure** **3**A), we isolated OT‐1 T cells from the splenocytes of OT‐1 mice using magnetic beads and labeled them with carboxyfluorescein succinate (CFSE). In addition, BMDCs were isolated and cultured from mouse bone marrow and treated with GP‐OVA_257‐264_ for 24 h. Labeled OT‐1 cells were then cocultured with antigen‐loaded BMDCs in vitro at a ratio of 10:1 for 72 h, and T cell proliferation was assessed by the FACS dilution rate of CFSE. The results showed that GP‐OVA_257‐264_ could induce a CD8^+^ T cell proliferation rate of more than 50%, whereas the rate induced by GP and the other controls was less than 7% (Figure 3B). For the in vivo experiment (Figure 3C), splenocytes from OT‐1 mice were first labeled with CFSE and then intravenously injected into C57BL/6 mice, which were inoculated the next day with GP‐OVA_257‐264_ and controls, and the proliferation rate of OT‐1 CD8^+^ T cells was detected by flow cytometry on the 3rd‐day post‐immunization. The results showed that GP‐OVA_257‐264_ induced a 32.4% CD8^+^ T cell proliferation rate in vivo, whereas GP and the other controls induced less than 0.7% proliferation (Figure 3D). Free OVA_257‐264_ can also induce the activation of OT‐1 cells for direct loading onto MHC‐I. GP‐OVA_257‐264_ effectively induced the specific proliferation of OT‐1 transgenic CD8^+^ T cells.

Subsequently, we further explored the ability of GP‐OVA_257‐264_ to activate antigen‐specific CD8^+^ T cells in wild‐type C57BL/6 mice. The mice were subcutaneously inoculated with GP‐OVA_257‐264_ or control every 14 days, and the splenocytes were isolated for FACS analysis 7 days after the second immunization. Tetramer staining showed that the proportion of MHC‐I‐OVA_257‐264_ tetramer^+^ CD8^+^ T cells in the GP‐OVA_257‐264_‐inoculated mice was significantly increased, reaching 2.8%, which is 5 times greater than that in the OVA_257‐264_‐inoculated mice (Figure 3E,F; Figure S15, Supporting information). FACS further showed that in the GP‐OVA_257‐264_‐inoculated mice, the proportion of naïve CD8^+^ T cells (CD44^−^ CD62L^+^) was reduced from 62.3% to 54.6% (Figure 3G), and the proportion of central memory CD8^+^ T cells (CD44^+^ CD62L^+^) increased from 22.8% to 29.4% (Figure 3H). Central memory CD8^+^ T cells have a more ideal antitumor ability in vivo, and their increase may be helpful to improve the quality of the cellular immune response.^[^ ^18^ ^]^ IFN‐*γ*‐specific ELISpot analysis showed that when mouse splenocytes were restimulated with OVA_257‐264_ in vitro, GP‐OVA_257‐264_‐inoculated mice produced a large number of IFN‐*γ*‐specific spots (up to 170 per 10^5^ splenocytes), whereas control groups, including free OVA_257‐264_, GPs, and PBS, minimally induced the production of IFN‐*γ*‐specific spots (Figure 3I). These results further showed that GP‐OVA_257‐264_ can efficiently induce mice to produce antigen‐specific CD8^+^ T cells.

Furthermore, we explored the killing capacity of GP‐OVA_257‐264_‐induced antigen‐specific CD8^+^ T cells through lysis experiments with target cells in vivo. The splenocytes from C57BL/6 mice were divided into two equal parts. One part was pulsed with OVA_257‐264_ peptide and then labeled with a high concentration (5.0 µM) of CFSE as target cells (CFSE^high^), and the other part was labeled with a low concentration (0.5 µM) of CFSE as control cells (CFSE^low^). The two fractions were mixed together (1:1) and transferred intravenously into C57BL/6 mice inoculated with GP‐OVA_257‐264_ and other controls 7 days ago, and the ratio of CFSE^high^ and CFSE^low^ splenocytes was measured by FACS 18 h later (Figure 3J). The results showed that in the PBS control group, the target cells were not killed, and the population of CFSE^high^ and CFSE^low^ cells was completely the same. In the GP‐OVA_257‐264_ group and OVA_257‐264_ peptide group, 24.5% and 3.2% of the target cells were specifically lysed, respectively (Figure 3K,L). These results provide good evidence that GP‐OVA_257‐264_ can efficiently induce the production of antigen‐specific T cells and specifically lyse antigen‐positive target cells.

In addition, we also examined the ability of GPs as a vector to induce specific humoral immune responses against peptide antigens. Here, we selected the MHC‐II‐restricted antigen OVA_323‐339_ (ISQAVHAAHAEINEAGR, from protein OVA) as the model antigen^[^ ^19^ ^]^ and coupled it to the GPs to construct vaccine particles of GP‐OVA_323‐339_ (Figure S16, Supporting Information). Then, GP‐OVA_323‐339_ along with free OVA_323‐339_ peptide and OVA protein (in the same molar dose as OVA_323‐339_) as controls were injected into the inguinal area of C57BL/6 mice every 14 days. At Day 7 post each immunization, the titer of OVA‐specific immunoglobulin G (IgG) and the subtypes IgG1 and IgG2a in serum were detected by ELISA. The results showed that the titers of IgG, IgG1, and IgG2a in GP‐OVA_323‐339_‐inoculated mice were much higher than those in OVA_323‐339_‐inoculated mice (Figure 3M–O, Figure S17, Supporting Information). It is worth mentioning that the titers of these antibodies were even higher than those of the OVA whole protein group, especially for IgG2a. The humoral immune response induced by GPs has an obvious T_H_1 bias. GP carriers can stimulate the body to produce high‐intensity T_H_1‐biased antibody immune responses against conjugated peptides, which shows important application potential in many fields.

**Figure 4:**
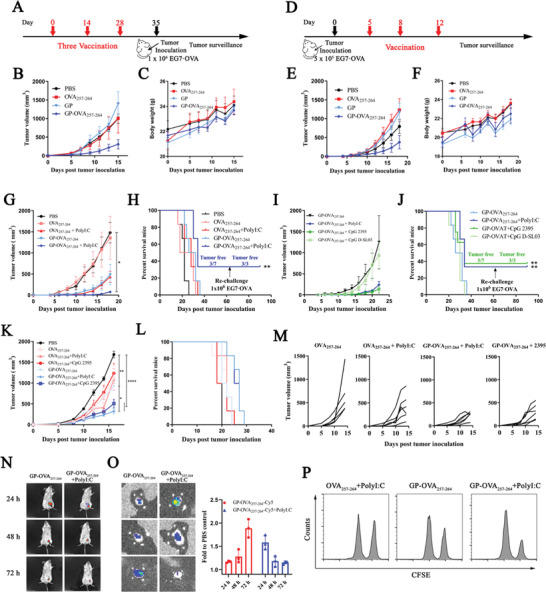
Antitumor activity of GP‐OVA_257‐264_ in combination with TLR agonists in the EG7·OVA tumor model. A–C) Antitumor activity of GP‐OVA_257‐264_ in the prophylactic model. Schematic diagram of the prophylactic tumor model (A), in which the mice are inoculated thrice subcutaneously every 14 days. 7 days after the third immunization, the mice are challenged with 1 × 10^6^ EG7·OVA lymphoma cells. B) Tumor growth and C) body weight profiles in GP‐OVA_257‐264_‐ and control‐treated mice (*n* = 7) are shown. D–F) Antitumor activity of GP‐OVA_257‐264_ in the therapeutic model. Schematic diagram of the therapeutic tumor model (D), in which the mice are inoculated with 5 × 10^5^ EG7·OVA cells first and inoculated thrice with different vaccines at Days 5, 8, and 12 post tumor inoculation. E) Tumor growth and F) body weight profiles in GP‐OVA_257‐264‐_ and control‐treated mice (*n* = 7) are shown. G–J) Antitumor activity of GP‐OVA_257‐264_ combined with TLR agonists in the prophylactic model. G) Tumor growth profiles and H) survival rate of mice (*n* = 7) inoculated with OVA_257‐264_ or GP‐OVA_257‐264_ combined with PolyI:C. Tumor growth profiles (I) and survival rate (J) of mice inoculated with GP‐OVA_257‐264_ with different TLR agonists (*n* = 7). K–M) Antitumor activity of GP‐OVA_257‐264_ combined with TLR agonists in a therapeutic model. Tumor growth profiles (K) and survival rate (L) in OVA_257‐264‐_ and GP‐OVA_257‐264_ combined with PolyI:C‐ or CpG 2395‐treated mice (n = 7) and tumor curve of a single mouse until Day 14 (M). N,O) Live imaging of the location and migration of GP‐OVA_257‐264_ combined with PolyI:C. BALB/c mice are inoculated with GP‐OVA_257‐264_‐Cy5 combined with or without PolyI:C, and the fluorescence images of inoculated mice (N), draining lymph nodes, and relative fluorescence intensity of Cy5 in draining lymph nodes compared to PBS inoculated mice at different time points (O) are shown. The black arrow in (N) represents the injection site. K) FACS histogram images of OVA‐specific CD8^+^ T cell lysis in vivo. Data represent the mean ± SEM. Statistical significance is calculated by one‐way ANOVA with Tukey's significant difference multiple comparisons. * *p* < 0.05, ** *p* < 0.01, *** *p* < 0.001, **** *p* < 0.0001.

Next, we explored the tumor inhibitory ability of GP‐OVA_257‐264_ through in vivo mouse tumor models. Prophylactic and therapeutic lymphoma models inoculated with EG7·OVA cells that have high expression of OVA protein were adopted. In the prophylactic model, C57BL/6 mice were inoculated with GP‐OVA_257‐264_ in the groin every 14 days and then subcutaneously injected with 1 × 10^6^ EG7·OVA lymphoma cells at Day 7 after the third immunization (**Figure** **4**A). As shown in Figure 4B, at Day 15 post tumor inoculation, the tumor volume of the PBS control group, or the mice inoculated with free OVA_257‐264_ or GPs all exceeded or was close to 1000 mm^3^. However, the tumor volume of GP‐OVA_257‐264_‐inoculated mice was much smaller with an average of less than 300 mm^3^. Simultaneously, the mouse body weight remained stable (Figure 4C), indicating that vaccine immunization has no obvious side effects. When the total dose of GPs remained unchanged, the inhibitory effect of GP‐OVA_257‐264_ on tumor growth was gradually enhanced with the increase in the equivalent of OVA_257‐264_ coupled to GPs, and the body weight also remained stable (Figure S18, Supporting Information). In the therapeutic model (Figure 4D), C57BL/6 mice were first subcutaneously inoculated with 5 × 10^5^ EG7·OVA lymphoma cells followed by immunization with GP‐OVA_257‐264_ or other controls on Days 5, 8, and 12. As shown in Figure 4E, GP‐OVA_257‐264_ also significantly inhibited tumor progression in tumor‐established mice, whereas free OVA_257‐264_ and GPs had minimal ability to inhibit tumor progression. In these treatment groups, mice also had stable body weights (Figure 4F) and showed little evidence of systemic hepatotoxicity, nephrotoxicity, and pathological changes in major organs (Figure S19, Supporting Information). These results show that GP‐OVA_257‐264_ can significantly inhibit tumor growth in a mouse model.

Next, we explored the combination of GP‐OVA_257‐264_ with immune adjuvants to further enhance tumor inhibition activity. We first tested the TLR3 agonist PolyI:C.^[^ ^20^ ^]^ In the constructed protective EG7·OVA subcutaneous lymphoma model, the combined immunization of GP‐OVA_257‐264_ and PolyI:C significantly improved the tumor inhibition efficiency. At 15 days post‐tumor inoculation, the tumor growth of the mice was almost completely inhibited; 3/7 mice had complete tumor remission up to 60 days of the observation period. For the mice whose tumors were completely suppressed, we reinoculated them with 1 × 10^6^ EG7·OVA lymphoma cells 2 months later, and all the mice completely rejected the growth of tumors in the following long‐term observation period (Figure 4G,H). Then, we further tested CpG D‐SL03 and CpG 2395,^[^ ^21^ ^]^ both of which are c‐type CpG ODN adjuvants modified with all phosphorus‐sulfur bonds containing palindromic sequences. The former contains the aacgtt palindrome motif (5′‐tcgcgaacgttcgccgcgttcgaacgcgg‐3′), whereas the latter contains the cggcgc palindromic motif (5′‐tcgtcgttttcggcgc:gcgccg‐3′). In the prophylactic EG7·OVA lymphoma model, CpG D‐SL03 had no synergistic effect with GP‐OVA_257‐264_, but CpG 2395 also significantly enhanced the tumor inhibition of GP‐OVA_257‐264_ at levels similar to that noted for PolyI:C (Figure 4I,J). Furthermore, we constructed a therapeutic EG7·OVA model to explore the inhibitory effect of GP‐OVA_257‐264_ combined with adjuvants on the growth of pre‐established tumors. The results showed that the tumor volume in the OVA_257‐264_ + PolyI:C or OVA_257‐264_ + CpG 2395 group was greater than 1000 mm^3^ on Day 15 post tumor inoculation, whereas that in the GP‐OVA_257‐264_ + PolyI:C or GP‐OVA_257‐264_ + CpG 2395 group was less than 500 mm^3^ (Figure 4K,M). In addition, these combined immunizations did not cause side effects of systemic cytokine release (Figure S20, Supporting Information). Immunization with peptides and adjuvants such as OVA_257‐264_ + PolyI:C is a commonly used immunization strategy in clinical trials, whereas the combined immunization of GP‐OVA_257‐264_ with PolyI:C or CpG 2395 showed greatly improved tumor inhibition and induced long‐term antitumor rejection.

Next, we preliminarily explored the mechanism of the enhanced antitumor activity of GP‐OVA_257‐264_ by PolyI:C and CpG 2395 adjuvant. When Cy5 labeled GP‐OVA_257‐264_‐Cy5 particles were injected into the inguinal area of ​​C57BL/6 mice together with PolyI:C (Figure 4N,O), the fluorescence intensity in draining lymph nodes peaked at 24 h post‐injection with no fluorescence noted in all other organs (Figure S21, Supporting Information). In contrast, the fluorescence intensity peaked after 72 h in the group of GP‐OVA_257‐264_ alone. We also found that coimmunization with CpG 2395 induced mild swelling of the spleen and draining lymph nodes (Figure S22, Supporting Information). These results may imply that the combination with PolyI:C or CpG 2395 can significantly increase the immune activation of the spleen and lymph nodes and improve the migration efficiency of GP‐OVA_257‐264_, thereby inducing stronger immune activation. The in vivo target cell killing experiment showed that (Figure 4P) the combined immunization of GP‐OVA_257‐264_ and PolyI:C significantly increased the target cell lysis efficiency from 25% to approximately 50%. Tetramer staining experiments showed that (Figure S23, supporting information), after three immunizations, the combined immunization of GP‐OVA_257‐264_ and CpG 2395 induced 2.95% OVA‐tetramer^+^ CD8^+^ T cells, whereas the strategy of single GP‐OVA_257‐264_ or peptide combined with adjuvant (OVA_257‐264_ + PolyI:C) used in the clinical trial only produced approximately 1.13% and 0.6% positive cells, respectively. These results preliminarily explain and confirm why the combination of immune adjuvants enhances the tumor treatment ability of GP‐OVA_257‐264_.

**Figure 5:**
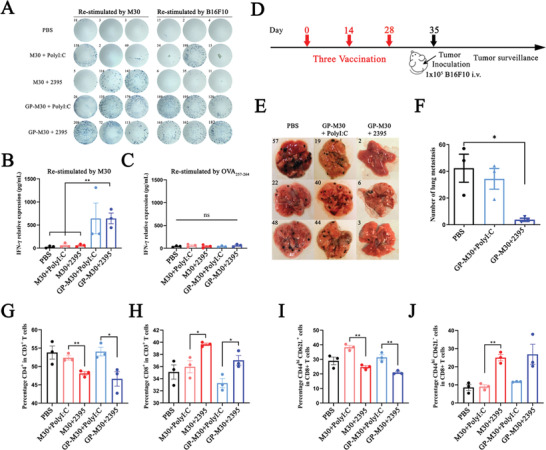
GP‐M30 induced B16F10 neoantigen M30‐specific cellular immune responses and antitumor activation in a B16F10 melanoma model. A–C) IFN‐*γ* ^+^ ELISpot and ELISA of M30‐specific CD8^+^ T cells. C57BL/6 mice are inoculated twice with M30 or GP‐M30 combined with PolyI:C or CpG 2395. One part of the splenocytes of inoculated mice is restimulated with M30 or B16F10 cells ex vivo and assessed by ELISpot (A), and another part is restimulated with M30 (B) or OVA_257‐264_ (C) ex vivo and assessed by ELISA. D–F) Antitumor activity in a prophylactic lung metastatic B16F10 melanoma model. Schematic diagram of the prophylactic lung metastatic B16F10 melanoma model (D), in which the mice are inoculated three times subcutaneously every 14 days. On Day 7 after the third immunization, the mice are intravenously challenged with 1 × 10^5^ B16F10 cells. The mice are euthanized 20 days post‐tumor challenge, and images of lung (E) and number of B16F10 lung metastases (F) in different mice are illustrated. G–J) FACS analysis of T cell differentiation in the splenocytes of inoculated mice in (D–F). The differentiation proportion of T cells to CD4^+^ T (G) or CD8^+^ T (H) cells and the differentiation proportion of CD8^+^ T cells to TEM (I) or TCM (J) are shown. Data represent the mean ± SEM. Statistical significance is calculated by one‐way ANOVA with Tukey's significant difference multiple comparisons. * *p* < 0.05, ** *p* < 0.01.

Preliminary experiments verified that the combination of GP‐OVA_257‐264_ particles with PolyI:C and CpG 2395 has a very ideal tumor suppressing ability. To this end, we next evaluated the antitumor ability of this system in more tumor models. We selected neoantigen peptides of different types of tumors to prepare GP‐Neoantigen vaccine particles and constructed corresponding syngeneic mouse tumor models to verify the inhibitory effect of the combined immunization of GP‐Neoantigen particles and adjuvant on tumor growth. We first selected B16F10 melanoma for evaluation. The vaccine particles GP‐M30 were prepared by conjugating GPs with B16F10 melanoma‐specific neoantigen M30, and the HPLC results showed that the ligation reaction was very efficient (Figure S24, Supporting Information). Subsequently, we tested the ability of the combined immunization of GP‐M30 and adjuvant to induce an antigen‐specific CD8^+^ T cell response in mice. Here, C57BL/6 mice were inoculated with the vaccine particles once every 14 days, and the splenocytes were isolated at Day 7 post‐second immunization and restimulated in vitro with M30, B16F10 cells, or OVA_257‐264_ followed by detection of the proliferation of IFN‐*γ* ^+^ CD8^+^ T cells by ELISpot (**Figure** **5**A) and the secretion of IFN‐*γ* by ELISA (Figure 5B,C). Combination immunization of GP‐M30 with PolyI:C or CpG 2395 induces much more pronounced IFN‐*γ*‐specific puncta production and considerably greater IFN‐*γ* release compared to the M30 peptide. The immunization is M30 specific, whereas restimulation of OVA_257‐264_ cannot achieve this response.

Subsequently, we used a prophylactic B16F10 tumor model to examine the inhibitory effect of GP‐M30 on tumor growth. Here, C57BL/6 mice were inoculated every 14 days three times and then transferred with 1 × 10^5^ B16F10 cells through the tail vein at Day 7 after the last immunization and dissected to count the number of lung tumor metastases at Day 15 after tumor cell transfer (Figure 5D). The results revealed 57 tumor foci in the PBS group. In contrast, GP‐M30 + CpG 2395 significantly inhibited the formation of B16F10 lung metastases with less than 7 tumor foci identified, and the inhibition efficiency reached 90%. However, GP‐M30 + PolyI:C was not effective, and the number of tumor foci reached 44 (Figure 5E,F). CpG 2395 appears to show a stronger synergistic effect than PolyI:C in the B16F10 tumor model. To explore why CpG 2395 and PolyI:C have different synergistic effects, we examined CD8^+^ T cell activation in mice inoculated with these combinations. As shown in Figure 5G,H, in the splenocytes at Day 7 post‐second immunization from the CpG 2395 combined immunization group, the proportion of CD8^+^ T cells in total T cells increased from 35% to 39.4%, and CD4^+^ T cells decreased from 53.6% to 47.5%, but they were not significantly changed in the PolyI:C combined immunization group (Figure S25A,B, Supporting Information). Furthermore, the proportion of effector memory T cells in CD8^+^ T cells increased from 8.5% to 25.3% in the CpG 2395 combined immunization group. In contrast, the proportion of central memory T cells increased from 29.1% to 38.5% in the PolyI:C combined immunization group (Figure 5I,J; Figure S25C, D, Supporting Information). These results seem to indicate that CpG 2395 and PolyI:C, which represent different types of TLR ligands, may have different mechanism for activating tumor immunity. The former seems to be more inclined to enhance the differentiation of T cells into CD8^+^ T cells, whereas the latter is more advantageous in inducing an enhanced proportion of central memory T cells.

**Figure 6:**
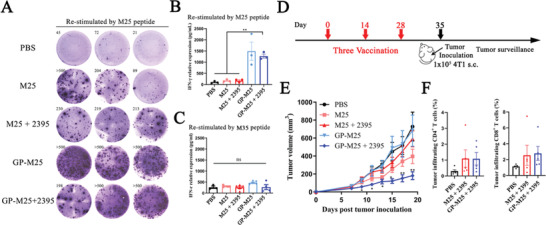
GP‐M25 induced 4T1 neoantigen‐specific cellular immune responses and antitumor activation in a 4T1 breast cancer model. A‐C) IFN‐*γ* ^+^ ELISpot and ELISA of M25‐specific CD8^+^ T cells. BALB/c mice are inoculated twice with M25 or GP‐M25 combined with or without CpG 2395. One part of the splenocytes of inoculated mice is restimulated with M25 ex vivo and assessed by ELISpot (A), and another part is restimulated with B) M25 or C) M35 and assessed by ELISA. D‐F) Antitumor activity in prophylactic 4T1 models. D) Schematic diagram of prophylactic 4T1 models, in which the mice are inoculated thrice subcutaneously every 14 days with different vaccines. On Day 7 after the last immunization, the mice are challenged with 1 × 10^5^ 4T1 cells. Tumor growth profile (E) and the proportions of CD4^+^/CD8^+^ T cells in CD45^+^ tumor‐infiltrating lymphocytes (F) are illustrated (*n* = 5). Data represent the mean ± SEM. Statistical significance is calculated by one‐way ANOVA with Tukey's significant difference multiple comparisons, * in (D) representative versus M25. * *p* < 0.05, ** *p* < 0.01.

Then, we further examined the tumor therapeutic capacity of the GP‐Neoantigen system in a 4T1 breast cancer model. As previously described, we first conjugated GP with the 4T1 breast cancer‐specific neoantigen M25 to construct GP‐M25 vaccine particles, and the HPLC results showed that the reaction was quite efficient (Figure S26, Supporting Information). Then, we examined the specific cellular immune activation of the vaccine particles in mice. Animal immunization of BALB/c mice and subsequent ELISpot and ELISA analyses were similarly performed. Another 4T1‐specific neoantigen, M35, was used as a control. The results showed that GP‐M25 immunization produced more ELISpot spots and secreted higher amounts of IFN‐*γ* than M25 immunization; however, combination with CpG 2395 showed no further improvement (**Figure** **6**A,B). Stimulation of these splenocytes with M35 does not produce IFN‐*γ* (Figure 6C), showing that the cellular immunity induced is specific. Subsequently, we used a prophylactic 4T1 syngeneic xenograft model to explore the tumor inhibition effect of GP‐M25 (Figure 6D). The results showed that GP‐M25 + CpG 2395 showed a much stronger tumor growth inhibitory function. On Day 19 post tumor inoculation, the tumor volume in GP‐M25 + CpG 2395‐inoculated mice was less than 200 mm^3^, and the tumor volume in the GP‐M25 alone group was also reduced. In contrast, the tumor volume in M25‐ or M25 + CpG 2395‐inoculated mice was already greater than 600 mm^3^ (Figure 6E). Furthermore, we found that combined immunization with GP‐M25 + CpG 2395 significantly increased the numbers of CD8^+^ T cells and CD4^+^ T cells that infiltrated into the tumors of the treated mice (Figure 6F), which may represent a mechanism for the vaccine to improve the tumor inhibition ability of the body.

**Figure 7:**
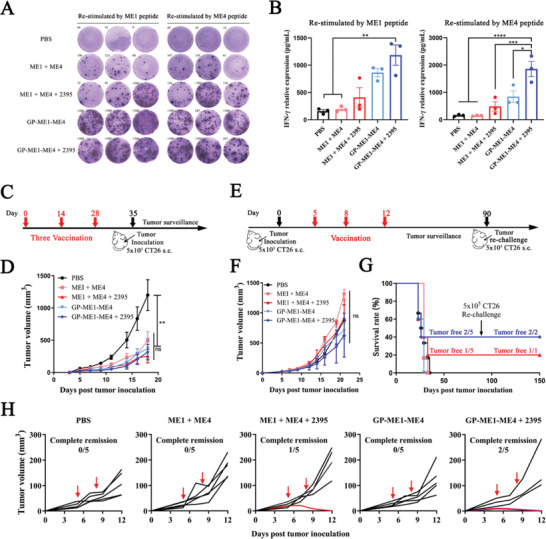
GP‐ME1‐ME4 induced CT26 neoantigen‐specific cellular immune responses and antitumor activation in the CT26 colon cancer model. A,B) IFN‐*γ* ^+^ ELISpot and ELISA of ME1/ME4‐specific CD8^+^ T cells. BALB/c mice are inoculated twice with ME1 + ME4 or GP‐ME1‐ME4 combined with or without CpG 2395. The splenocytes of inoculated mice are restimulated with ME1 or ME4 neoantigens ex vivo, and the proliferation of IFN‐*γ* ^+^ CD8^+^ T cells is detected by A) ELISpot and B) ELISA. C,D) Antitumor immunity in prophylactic CT26 models. Schematic diagram of prophylactic CT26 models (C), in which the mice are inoculated thrice subcutaneously every 14 days, and the mice are challenged with 1 × 10^5^ CT26 cells 7 days after the last immunization. The tumor growth profile (D) is illustrated (*n* = 5). E–H) Antitumor immunity in therapeutic CT26 models. Schematic diagram of the therapeutic tumor model (E), in which the mice are inoculated with 5 × 10^5^ CT26 cells first and inoculated thrice subcutaneously at Days 5, 8, and 12 post tumor inoculation. Overall tumor growth profiles (F), survival and tumor‐free rate (G), and tumor growth profiles of different vaccines (H) are illustrated. Red arrows indicate the time point of vaccination. Data represent the mean ± SEM. Statistical significance is calculated by one‐way ANOVA with Tukey's significant difference multiple comparisons, * *p* < 0.05, ** *p* < 0.01, *** *p* < 0.001, **** *p* < 0.0001.

Next, we further tested the tumor therapeutic ability of this system in a colon cancer model. As previously described, we conjugated GPs simultaneously with two colorectal cancer cell CT26‐specific neoantigens, ME1 and ME4, to obtain GP‐ME1‐ME4 particles. HPLC results showed that the coupling was very efficient (Figure S27, Supporting Information). Subsequently, similar to the previous process, we examined the specific cellular immune activation induced by GP‐ME1‐ME4 combined with CpG 2395 in BALB/c mice through ELISpot and ELISA. GP‐ME1‐ME4 induced stronger ME1‐ or ME4‐specific cellular immune responses than ME1 + ME4 or ME1 + ME4 + CpG 2395 (**Figure** **7**A,B). In the prophylactic CT26 syngeneic subcutaneous xenograft model (Figure 7C), immunization with GP‐ME1‐ME4 + CpG 2395 also showed good tumor inhibition. On Day 18 post tumor inoculation, the tumor volume of mice was less than 400 mm^3^, whereas that of the PBS control group exceeded 1000 mm^3^. In addition, and immunization with GP‐ME1‐ME4 alone also had a good tumor inhibitory effect (Figure 7D). In the tumor‐established therapeutic CT26 syngeneic tumor model (Figure 7E), GP‐ME1‐ME4 + CpG 2395 immunization achieved complete tumor clearance in 40% of the mice; 20% of the mice in the free ME1 + ME4 + CpG 2395 immunization group also obtained complete tumor clearance (Figure 7F–H), which may indicate that ME1 and ME4 neoantigen epitopes have relatively stronger immune activation ability. However, in the treatment group, the individual differences in the tumors of the mice were relatively large, and the remaining 3/5 mice had rapid tumor growth, which was reflected in the fact that the overall average tumor volume showed minimal differences compared with that of the PBS group (Figure 7F). Consistent with the previous experimental results, three months later, these tumor‐cleared mice were reinoculated subcutaneously with 5 × 10^5^ CT26 cells, and 100% of the mice exhibited no tumor growth. Further improving the effect to achieve tumor clearance in all mice must be addressed in the next stage.

## 3 Conclusion

In conclusion, we have developed a novel tumor vaccine system GP‐Neoantigen based on yeast polysaccharide shell particles, which can stimulate the body to produce a strong antigen‐specific CD8^+^ T cell immune response against various neoantigen peptides and thus be used for effective tumor treatment. Compared with other synthetic nanoparticle systems, this system is relatively simple to prepare and stable between batches with a uniform particle size and extremely high specificity for uptake by APCs, including DCs and macrophages. This particle system showed strong immune activation ability in animal models and effectively inhibited the growth of tumors in various mouse syngeneic tumor models, such as the EG7·OVA lymphoma model, B16F10 melanoma, 4T1 breast cancer, and CT26 colon cancer. Combined immunization with PolyI:C or CpG 2395 adjuvant further significantly enhanced the tumor inhibitory effect. Of note, this strategy can achieve complete tumor eradication in multiple mouse models and induce long‐term tumor rejection memory in mice with tumor eradication, completely avoiding growth of the reinoculation tumor. These results provide broad possibilities for its further clinical promotion and personalized vaccine therapy.

## 4 Experimental Section

All materials, methods, and additional data can be found in the Supporting Information. All animal experiments were performed in accordance with the National Institute of Health Guidelines under protocols approved by the Animal Ethical and Welfare Committee of Nankai University (2019‐SYDWLL‐000413). The mouse lymphoma cell line (EG7·OVA), melanoma cell line (B16F10), breast cancer cell line (4T1), colon cancer cell line (CT26), human fibroblast cell line (293T), human liver cell line (LO2) and mouse embryonic fibroblast cell line (NIH‐3T3) were obtained from American Type Culture Collection (ATCC).

## Conflict of Interest

The authors declare no conflict of interest.

## Supporting information

Supporting Information

## Data Availability

The data that support the findings of this study are available from the corresponding author upon reasonable request.
