# Supplementary material for: A Potent Micron Neoantigen Tumor Vaccine GP‐Neoantigen Induces Robust Antitumor Activity in Multiple Tumor Models
Source: Adv Sci (Weinh). 2022 Jun 16;9(24):2201496. doi: 10.1002/advs.202201496 (PMC9403634; doi:10.1002/advs.202201496)
Supplement: Supplementary file 1 — Supporting Information [file ADVS-9-2201496-s001.pdf]

ADVANCED SCIENCE
Open Access

Supporting Information

for *Adv. Sci.*, DOI 10.1002/advs.202201496

A Potent Micron Neoantigen Tumor Vaccine GP-Neoantigen Induces Robust Antitumor Activity in Multiple Tumor Models

Zhe Jing, Shuqing Wang, Keyuan Xu, Qian Tang, Wenjing Li, Wei Zheng, Haobo Shi, Kailing Su, Yanting Liu and Zhangyong Hong**

Supporting Information

A potent micron neoantigen tumor vaccine GP-Neoantigen induces robust antitumor activity in multiple tumor models

Zhe Jing, Shuqing Wang, Keyuan Xu, Qian Tang, Wenjing Li, Wei Zheng, Haobo Shi, Kailing Su, Yanting Liu, Zhangyong Hong**

Methods and materials

Animals: C57BL/6 mice, BALB/c mice and OT1 transgenic mice (C57BL/6-Tg(Tcra Tcrb)1100Mjb/J) with TCRs specific for the peptide OVA257-264 (SIINFEKL) were provided by Beijing Vital River Laboratory Animal Technology Co., Ltd. (Beijing, China). All mice were raised under specific pathogen-free conditions in the Experimental Animal Center of Nankai University. All studies were approved by the Nankai University Animal Committee.

In vitro stimulation and in vivo immunization dose: For in vitro stimulation, 50 μg mL^{-1} GPs or GP-$OVA_{257-264}$ or 1.0 nmol mL^{-1} $OVA_{257-264}$ were used. For in vivo immunization, mice were administered 20 nmol antigen peptide or 1.0 mg antigen peptide-conjugated GPs subcutaneously with or without 50 μg PolyI:C (Invivogen, CA, USA) or 50 μg CpG ODN 2395 (Invivogen, CA, USA) in 100 μl of sterile PBS. For the preparation of peptide-conjugated GPs, 20 nmol $OVA_{257-264}$, $OVA_{323-339}$ or neoantigen was conjugated to 1.0 mg GPs.

Preparation of glucan particles (GPs): Briefly, 40 g Saccharomyces cerevisiae powder (Angel Yeast, Hubei, China) was resuspended in double distilled water (ddH_2O), washed thrice with ddH_2O, and centrifuged at 2000 rpm for 5 minutes each time. Then, the precipitate was resuspended in 1 M NaOH solution and stirred at 80 ℃ for 1 hour. The precipitate was washed thrice with ddH_2O and centrifuged at 2000 rpm for 5 minutes each time. The pH value was adjusted to 4-5 with 1 M hydrochloric acid, and the suspension was stirred at 55 ℃ for 1 hour and then centrifuged at 3500 rpm for 5 minutes. Later, the precipitate was washed thrice with ddH_2O and centrifuged at 3500 rpm for 5 minutes each time followed by

resuspension with isopropanol, five washes with isopropanol and centrifugation at 3500 rpm for 2 minutes each time. The precipitate was then suspended in acetone, washed twice with acetone and centrifuged at 3500 rpm for 2 minutes each time. Finally, the obtained glucan particles (GPs) were dried naturally in a fume hood, dispersed in a mortar, and stored at room temperature under dry conditions.

Preparation of GP-Neoantigen particles: Glucan particles (GPs) were resuspended in 0.5 M sodium hydroxide solution and stirred at room temperature for thirty minutes followed by the addition of the same mass of N_3-epoxy linker. The solution was stirred at room temperature overnight. The mixture was centrifuged at 5000 rpm for 3 minutes, and the supernatant was discarded. The precipitate was resuspended in ddH_2O, and the pH was adjusted to 2.5 with hydrochloride to stop the reaction. Then, the precipitate was washed thrice with ddH_2O and further washed thrice with DMSO through centrifugation and resuspension. Finally, the pellets were resuspended in DMSO to obtain a 25 mg mL^{-1} GP-N_3 solution. The synthesized peptides $OVA_{257\text{-}264}$, $OVA_{323\text{-}339}$, 4T1-M25, CT26-ME1, CT26-ME4, and B16-M30 (GL Biochem, Shanghai, China) with a free C-terminal cysteine residue were dissolved in DMSO to a 10 mM solution. DBCO-PEG4-maleimide (Sigma Aldrich, MO, USA) was also dissolved in DMSO to yield a 10 mM solution. The equivalent ratio of 1.1:1 cysteine-peptide and DBCO-PEG4-maleimide was added together, and then 0.5% (v/v) N,N-diisopropylethylamine (DIPEA) was added. The reaction was allowed to stand at 25 ℃ for 2 hours, and the reaction efficiency was measured by high-performance liquid chromatography (HPLC). After the reaction was complete, the obtained DBCO-peptide was precipitated with ether and dried naturally, and the precipitate was redissolved in DMSO. GP-N_3 and DBCO-peptide were simply mixed according to the required loading capacity and stirred at room temperature for 2 hours, and the supernatant was measured by HPLC to monitor the reaction efficiency. Finally, DMSO was completely removed from the reaction solution by centrifugation and resuspension with ddH_2O followed by five PBS washes.

Fluorescence labeling of GPs and DBCO-$OVA_{257\text{-}264}$: Rhodamine B (RhoB, Sigma Aldrich), FITC (Sigma Aldrich) and Cy5 (MedChemExpress, NJ, USA) were dissolved to 100 mM with DMSO. For labeling of GPs, 500 mg GPs were resuspended in PBS to 20 mg mL$^{-1,}$ and the pH was adjusted to 8.5. Then, 2 μL fluorochrome was added and stirred at room temperature for one hour. Then, the pellets were washed five times with ddH_2O to remove excess fluorochrome. For labeling of $OVA_{257\text{-}264}$ and DBCO-$OVA_{257\text{-}264}$, peptides were

dissolved to 10 mM with DMSO. An equivalent ratio of 1:2 peptides and fluorochrome was added together, and then 0.5% (v/v) DIPEA was added. The reaction was allowed to stand at room temperature for two hours. Acetone was added to precipitate the labeled peptide-FITC/RhoB/Cy5 or DBCO-peptide-FITC/RhoB/Cy5, and the precipitate was redissolved to 10 mM with DMSO. This step was repeated thrice. After acetone was completely volatilized, the precipitate was dissolved in DMSO and ready for a follow-up experiment. To obtain fluorescently labeled GP-$OVA_{257-264}$, GP-N_3 was reacted with DBCO-$OVA_{257-264}$-FITC/RhoB/Cy5 for 2 hours. DMSO was completely removed by centrifugation and resuspension with ddH_2O and PBS thrice.

Sample preparation for scanning electron microscopy and transmission electron microscopy: GP, GP-N_3 and GP-$OVA_{257-264}$ were suspended in ddH_2O and sonicated for 30 minutes to evenly disperse the particles. For TEM, the resulting solution was diluted to a suitable concentration with ddH_2O and added dropwise to the carbon support film on the copper net. The sample was dried naturally and observed by scanning electron microscopy (HITACHI HT7700 Exalens). For SEM, after ultrasonic dispersion, centrifugation at 3000 rpm for 5 minutes and removal of the supernatant, the GP/GP-N_3/GP-$OVA_{257-264}$ particles were successively soaked in 30%, 50%, 70%, 80%, 90%, and 100% (three times) ethanol solution for 15 minutes to completely dehydrate them. Then, the dehydrated GP, GP-N_3 and GP-$OVA_{257-264}$ were diluted with ethanol to an appropriate concentration and dropped onto a glass slide followed by drying naturally and observation by transmission electron microscopy (QUANTA 200, FEI company, USA) operating at 20 kV.

Generation of BMDCs and BMDMs: Bone marrow was removed from the femur and tibia and fibula of C57BL/6 mice under sterile conditions and pipetted to disperse into a single-cell suspension in RPMI 1640 medium (Gibco Life Technologies, CA, USA) containing 2% penicillin & streptomycin (Gibco Life Technologies). The cells were centrifuged at 1400 rpm for 7 minutes, resuspended in 2 mL of red blood cell lysate (Solarbio Life Science, Beijing, China) for 5 minutes, collected before adding 8 mL of sterile PBS to stop the lysis and centrifuged at 1400 rpm for 7 minutes. For differentiation of BMDCs, the collected naïve bone marrow cells were resuspended in 1640 complete medium containing 5% (v/v) heat inactivated fetal bovine serum (Biological Industries, Kibbutz, Israel), 1% (v/v) penicillin and streptomycin, 40 ng mL^{-1} GM-CSF (Sino Biological, Cat. no. 51048-MNAH), 20 ng mL^{-1} IL-4 (Sino Biological, Cat. no. 51084-MNAE) and 50 μM β-mercaptoethanol and cultured in a

sterile cell incubator at 37 ℃ and 5% CO_2 for 6 days. Every two days, half of the medium was carefully replaced with fresh medium. Care was taken to avoid shaking throughout the culture process. On Day 6, all suspended and semiadherent cells were collected and counted for subsequent experiments. For differentiation of BMDMs, the collected naïve bone marrow cells were resuspended in 1640 complete medium containing 5% (v/v) heat inactivated fetal bovine serum, 1% (v/v) penicillin & streptomycin, 10 ng mL^{-1} M-CSF (Sino Biological, Cat. no. 51112-MNAH) and 50 μM β-mercaptoethanol and cultured in a sterile cell incubator at 37 ℃ and 5% CO_2 for 7 days. At Day 3 and Day 5, half of the medium was carefully replaced with fresh medium. At Day 7, all cells were collected and counted for subsequent experiments.

Cellular uptake of GP-OVA$_{257-264}$ by BMDCs, BMDMs and other cells: BMDCs or BMDMs were cultured in 24-well cell culture plates with 10 mm diameter cell cover glass (NEST, Wuxi, China) at a density of 1×10^6 cells mL^{-1}, 500 μL per well (2×10^5 cells mL^{-1} for other normal cell lines and 1×10^6 cells mL^{-1} for neutrophils). Twelve hours later, GP-OVA$_{257-264}$-RhoB or OVA$_{257-264}$-RhoB was added to different wells and further incubated for 4 hours. Subsequently, the cells were washed with PBS and fixed in an aqueous 4% formaldehyde solution for 20 minutes. This step was followed by cell membrane staining with FITC-labeled anti-mouse CD11c antibody (Biolegend, CA, USA) or DIO (UE, Suzhou, China) for 15 minutes and nuclear staining with DAPI for 5 mins. After washing with PBS twice, confocal laser scanning microscopy (CLSM, Leica TCS SP5, Germany) was used to monitor the uptake of the GP-OVA$_{257-264}$-RhoB particles.

In vitro activation, cross-presentation and cytotoxicity assays of BMDCs and BMDMs: For the in vitro activation and cross-presentation assays, BMDCs or BMDMs were cultured in 24-well cell culture plates with 14 mm diameter cell cover glass (NEST, Wuxi, China) at a density of 1×10^6 cells mL^{-1}, 500 μL per well. Twelve hours later, PBS, OVA$_{257-264}$, GP and GP-OVA$_{257-264}$ were added to different wells and further incubated for 24 hours. Subsequently, the supernatant was collected to detect the expression of IL-6 and IL-12 p70 using an enzyme-linked immunosorbent assay (ELISA) kit (Biolegend). The cells were collected and washed with PBS twice and stained with FITC anti-mouse CD11c antibody or FITC anti-mouse CD11b and APC anti-mouse F4/80 antibody combined with fluorochrome labeled anti-mouse CD80, CD86, CD40, H-2Kb/H-2Db (MHC-I) and I-A/I-E (MHC-II) (Biolegend). The cells were analyzed using a fluorescence-activated cell sorter (FACS, Calibur II, BD Biosciences, San Jose, CA, USA). An MTT assay was used to analyze the in vitro

cytotoxicity of GP-peptide. In brief, BMDCs were seeded in 96-well plates at a concentration of 1×10^5 cells per well and then incubated with various gradient concentrations of $OVA_{257-264}$ or GP-$OVA_{257-264}$ for 24 hours. After removing particles in suspension, MTT (0.5 mg mL^{-1}) diluted in fresh cell medium was added to each well and incubated for an additional 4 hours at 37 ℃. After removing the supernatant, 100 μL of DMSO was added to fully dissolve the formazan crystals, and the absorbance at 490 nm was measured on a microplate reader (EXL-800; Bio-Tek, Winooski, VT, USA).

In vitro and in vivo OT-1-cell proliferation following GP peptide stimulation: For the in vitro study of the antigen-specific proliferation of $CD8^+$ T cells stimulated with GP-$OVA_{257-264}$ particles, OT-1 TCR-transgenic mice were used to isolate OT-1 T cells from spleens using the Easy-Sep™ Mouse Naïve $CD8^+$ T Cell Isolation Kit (Stem Cell Technologies, Vancouver, Canada) according to the manufacturer's instructions. The isolated cells were stained with 5 mM carboxy fluorescein succinyl ester (CFSE, Invitrogen, CA, USA). Briefly, isolated cells were resuspended in PBS (2×10^6 cells mL^{-1}) with 5 μM CFSE and incubated for 15 minutes at 37 ℃. The labeling was quenched by adding an equal volume of PBS and incubating at 4 ℃ for 1-2 minutes, and the cells were washed with cold PBS to obtain CFSE-labeled OT-1 cells. In addition, BMDCs were treated with GP-$OVA_{257-264}$ to provide antigen-loaded DCs for experiments by incubation with GP-$OVA_{257-264}$ (50 μg mL^{-1} particles, loading of 1 μg mL^{-1} $OVA_{257-264}$) for 24 hours. Then, antigen-loaded BMDCs were cocultured with CFSE-stained OT1 $CD8^+$ T cells at a ratio of 1 to 10 in RPMI 1640 containing 10% (v/v) FBS, 2% (v/v) penicillin & streptomycin, and 50 μM β-mercaptoethanol in round-bottom 96-well plates for 72 hours. T cell proliferation was assessed by CFSE dilution assay with FACS Calibur. For the in vivo study of OT-1 $CD8^+$ T cell proliferation following stimulation with GP-$OVA_{257-264}$ particles, splenocytes from OT-1 transgenic mice were isolated and labeled with CFSE. Briefly, whole splenocyte cell suspensions were first prepared and purified from RBCs with RBC lysis solution. Then, the cells were labeled with CFSE as described above. CFSE-labeled OT-1 cells were intravenously injected into recipient C57BL/6 mice (1×10^7 cells per mouse), followed by immunization with PBS, $OVA_{257-264}$, GP and GP-$OVA_{257-264}$ on the second day. Three days post-immunization, recipient mice were euthanized, and the proliferation of $CD8^+$ T cells in the spleen (based on reduction in CFSE) was determined by flow cytometry.

In vivo localization and lymph node migration of GP-OVA$_{257\text{-}264}$: For live imaging localization, BALB/c mice were randomly grouped and subcutaneously immunized in the inguinal region with GP-OVA$_{257\text{-}264}$-Cy5 or OVA$_{257\text{-}264}$-Cy5. The fluorescence signals at the injection site and draining lymph nodes at 24 hours, 48 hours or 72 hours were monitored using an animal live imaging system (Lumina II, Xenogen, Waltham, USA). For in vivo antigen-presenting cell homing, C57BL/6 mice were randomly grouped and subcutaneously immunized in the inguinal with GP-OVA$_{257\text{-}264}$-FITC or controls. Seven days post-immunization, the mice were euthanized, and the draining lymph nodes were stained with APC anti-mouse CD11c (Biolegend), PE anti-mouse CD19 (Biolegend) or APC anti-mouse F4/80 (Biolegend) and analyzed by FACS Calibur.

In vivo assay of cellular immune responses: C57BL/6 mice (for OVA$_{257\text{-}264}$ or M30) or BALB/c mice (for 4T1-M25 or CT26-ME1, CT26-ME4) were randomly grouped and subcutaneously immunized in the inguinal twice every 14 days with GP-Neoantigen and controls. Seven days after the second vaccination, the mice were euthanized, and the splenocytes were obtained to detect cellular immune responses. For differentiation of memory T cells, splenocytes were stained with FITC anti-mouse CD62L and APC anti-mouse CD44 and analyzed by FACS Calibur. For OVA-specific cellular immune responses, splenocytes were stained with PE-conjugated H-2K^b-OVA tetramer (TS-5001-1C, MBL, Tokyo, Japan) for 30 minutes and FITC anti-mouse CD8 (Clone KT15, Santa Cruz Biotechnology, Texas, USA) for 20 minutes followed by washing with PBS twice and analysis by FACS Calibur. IFN-γ specific Elispot Kit (3321-2H/3321-2A, Mabtech, Sweden) to detect OVA- or neoantigen-specific cellular immune responses as described in the manufacturer’s guide. In brief, a MultiScreen-IP Filter Plate (MAIPS4510, Millipore, USA) was activated with 35% ethanol for 30 seconds one day in advance and then coated with 1.5 μg per well of coating antibody AN18 overnight. The next day, 5 $\times 10^5$ mouse splenocytes were added to the well, and 10 μM neoantigen peptides or 2 $\times 10^4$ tumor cells were added and coincubated at 37 ℃ for 48 hours. After incubation, the cells in the wells were removed. The plate was washed with PBS 5 times. Then, 100 μL of 1.0 μg mL^{-1} biotinylated detection antibody R4-6A2 was added to each well followed by incubation at room temperature for 2 hours and washing 5 times with PBS. For OVA$_{257\text{-}264}$ and M30, 100 μL streptavidin-HRP was added to each well. Then, TMB one-component color developing solution (Solarbio) was used for color development. For M25, ME1 and ME4, 100 μL streptavidin-ALP was added to each well, and BCIP-NBT (Sangon Biotech, Shanghai, China) was used for color development. IFN-γ spot-

forming cells (SFCs) were detected and analyzed using an ELISpot reader (AID iSpot, AID-Autoimmune Diagnostika GmbH, Strassberg, Germany).

In vivo CTL killing experiment: C57BL/6 mice were randomly grouped and immunized subcutaneously in the inguinal. At Day 7 post immunization, another untreated C57BL/6 mouse was euthanized. Its spleen was obtained under aseptic conditions, and the red blood cells were lysed to obtain a single cell suspension, which was divided equally into two parts. One part was incubated with 10 μM $OVA_{257\text{-}264}$ peptide in 1640 complete medium for 2 hours and then labeled with 5 μM CFSE ($CFSE^{high}$). The other half was incubated with only 1640 complete medium for 2 hours and then labeled with 0.5 μM CFSE ($CFSE^{low}$). Finally, the two parts of cells were mixed one-to-one and transferred back into the mice seven days post-immunization through the tail vein, with 1×10^7 total cells per mouse. At 18 hours post-transfer, the mice were euthanized, and the ratio of $CFSE^{low}$ and $CFSE^{high}$ cells in $CFSE^+$ ($CFSE^{low}$ plus $CFSE^{high}$) cells in splenocytes was measured by FACS Calibur.

$$\text{CTL killing rate} = [1- (\frac{CFSEhigh(\%)}{CFSElow(\%)} / \frac{CFSEhigh\ (PBS\ control,\%)}{CFSElow\ (PBS\ control,\%)})] \times 100\%$$

Detection of antigen-specific humoral immunity: C57BL/6 mice were randomly grouped and immunized subcutaneously in the inguinal every 14 days. At Day 7 post the second, third, and fourth immunizations, 50 μL of whole blood was collected from the marginal eye vein of the mice with an anticoagulant tube. After standing for 30 minutes at 4 ℃, serum was extracted by centrifuging at 3800 rpm for 10 minutes, and the antibody titer was detected by ELISA. In brief, 10 μg mL^{-1} OVA was coated onto microplates at 4 ℃ overnight. The plates were washed four times with PBS containing 0.05% Tween 20 (PBST) and blocked with 5% BSA solution at room temperature for 1 hour. After washing four times with PBST, serial gradient dilutions of serum were performed, and the plate was incubated at room temperature for 2 hours. The plates were washed four times with PBST and reacted with horseradish peroxidase (HRP)-labeled goat anti-mouse IgG (Sigma, St. Louis, MO, USA), IgG1 and IgG2a antibodies (Southern Biotech, Birmingham, USA) at room temperature for 1 hour. Finally, the plates were washed five times with PBST and developed with TMB substrate for 15 minutes. The absorbance at 450 nm and 570 nm was measured by a microplate reader (EXL-800, Bio-Tek). The IgG titer was defined as the maximum serum dilution (OD450 - OD570) value > 0.5. For the purpose of statistical analysis, a value of 25 was assigned for IgG titer < 50.

Tumor challenge and evaluation of antitumor activity: For the prophylactic grafted tumor model, C57BL/6 (for EG7 OVA and B16F10 models) or BALB/c (for 4T1 and CT26 models) mice were randomly grouped and immunized subcutaneously in the inguinal three times every 14 days with GP-Neoantigen and controls. Seven days after the last vaccination, the mice were subcutaneously challenged with tumor cells (1×10^6 EG7 OVA, 1×10^5 4T1, 5×10^5 CT26) in the right flank, and tumor volumes were measured with a caliper every 2 or 3 days according to length $\times$ width $\times$ width $\times$ 0.5. Mice with a tumor volume greater than 1500 mm^3 were considered dead. For the therapeutic grafted tumor model, C57BL/6 or BALB/c mice were challenged with tumor cells (5×10^5 EG7 OVA, 1×10^5 4T1, 5×10^5 CT26) in the right flank. After the tumor was visible, the mice were grouped by equal mean tumor volumes and immunized subcutaneously in the inguinal region at Day 5, Day 8, and Day 12 with GP-Neoantigen and controls. Then, tumor volumes were measured every 2 or 3 days, and mice with a tumor volume greater than 1500 mm^3 were considered dead. For the prophylactic pulmonary metastatic tumor model, C57BL/6 mice were randomly grouped and immunized subcutaneously in the inguinal three times every 14 days with GP-M30 combined with PolyI:C or CpG 2395. Seven days after the last vaccination, the mice were intravenously challenged with 1×10^5 B16F10 cells. Twenty days post-tumor challenge, the mice were euthanized, and the amount of black lung metastasis was counted.

Analysis of tumor infiltrating lymphocytes (TILs): Tumors were extracted and cut into small pieces in PBS. The tumor tissues were then oscillated digested in PBS with 50 U mL^{-1} collagenase IV (Invitrogen) for 2 hours at 37 ℃. Suspensions were filtered through a 40-μm cell strainer and washed thrice with PBS. The lymphocyte population was resuspended in 40% Percoll and gently dripped onto 80% Percoll. TILs were enriched by density gradient centrifugation at 1260 $\times$ g (speed up at level 5 and speed down at level 1) for 30 minutes at room temperature. After centrifugation, the white cell layer was collected and washed thrice with PBS followed by staining with PerCP anti-mouse CD45 and other fluorescently labeled antibodies and analysis by FACS.

Statistical analysis: Statistical analysis was performed using GraphPad PRISM 9.0.0 or OriginPro 8. Flow cytometry data were analyzed by FlowJo V10. All data are presented as the mean $\pm$ SEM except for special indications. The statistical significance of differences was analyzed with Student's t test or one-way ANOVA followed by a Tukey post-hoc test was carried out across groups by Statistical Product and Service Solutions, SPSS or GraphPad

PRISM 9.0.0. The log-rank test was used to compare survival differences for Kaplan–Meier plots using SPSS. In all cases, significance was defined as $p < 0.05$, * means $p < 0.05$, ** means $p < 0.01$, *** means $p < 0.001$, **** means $p < 0.0001$.

Table S1. HPLC elution program in GP-Neoantigen preparation.

Time（mins）	Solvent A	Solvent B	Monitor
0			Start
0.01	90%	10%	
25.00	0%	100%	Elution
35.00	0%	100%	
39.00	90%	10%	
40.00			Stop

Solvent A: 0.1% Trifluoroacetic in 90% ddH$_2$O and 10% acetonitrile.

Solvent B: 0.1% Trifluoroacetic in 10% ddH$_2$O and 90% acetonitrile.

Flow rate: 0.6 mL/min Wavelength: 220 nm Loading volume: 10 μL

Table S2. Image analysis of TEM images of GP, GP-N$_3$ and GP-OVA$_{257-264}$

**Particles**	**GP**	**GP-N$_3$**	**GP-OVA$_{257-264}$**
Counts	8	8	8
Long diameter (μm)	4.67 ± 0.49	5.09 ± 0.53	5.25 ± 0.42
Short diameter (μm)	3.54 ± 0.47	3.88 ± 0.32	4.41 ± 0.29

All values given as mean ± s.d.

Table S3. Amino acid sequences of the model antigen OVA and neoantigens.

Name	Amino acids sequence (from N to C)	Length (aa)
OVA$_{257-264}$	SIINFEKL	8
OVA$_{323-339}$	ISQAVHAAHAEINEAGR	17
B16-M30	PSKPSFQEFVDWENVSPELNSTDQPFL	27
4T1-M25	KDYTAAGFSSFQKLRLDLTSMQIITTD	27
CT26-ME1	LHSGQNHLKEMAISVLEARACAAAGQS	27
CT26-ME4	WKGGPVKIDPLALMQAIERYLVVRGYG	27

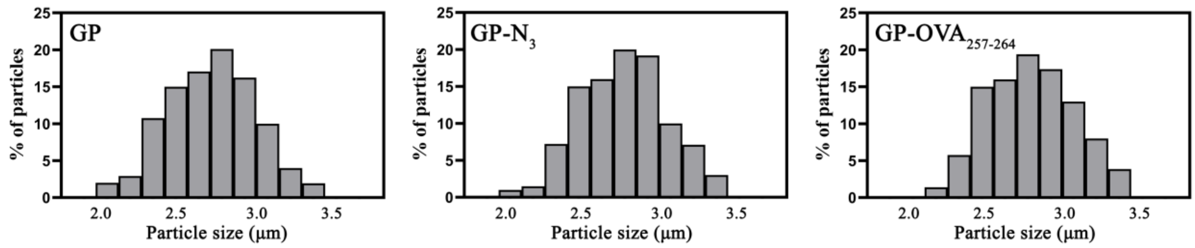

Figure S1. The statistical data histogram of particle size of GP, GP-N_3 and GP-$OVA_{257-264}$.

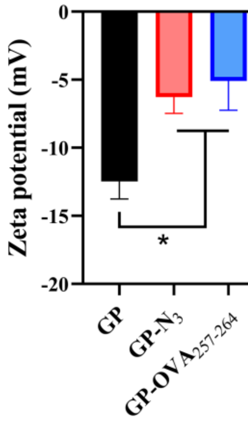

Figure S2. Zeta potential of GP, GP-N_3 and GP-$OVA_{257-264}$. Data represent the mean ± SEM. Statistical significance was calculated by one-way ANOVA followed by a Tukey post-hoc test across groups. n = 5, * $p < 0.05$.

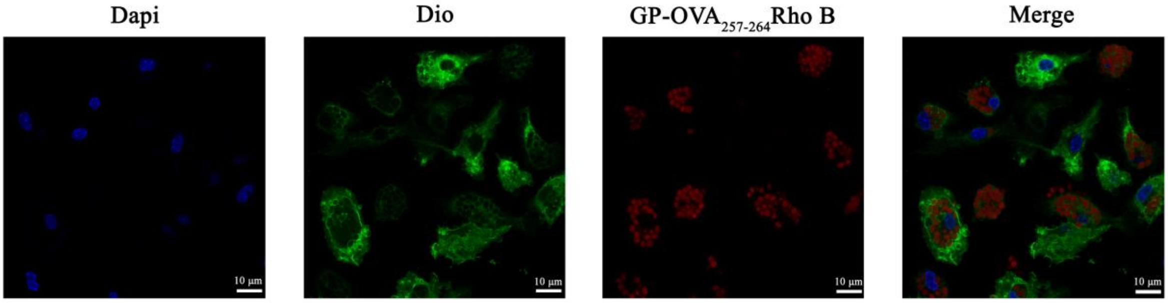

Figure S3. Cellular uptake of GP-$OVA_{257-264}$ particles by BMDMs. The cellular uptake of GP-$OVA_{257-264}$-RhoB (red signals) by BMDMs was analyzed by CLSM measurements at 24 hours of incubation.

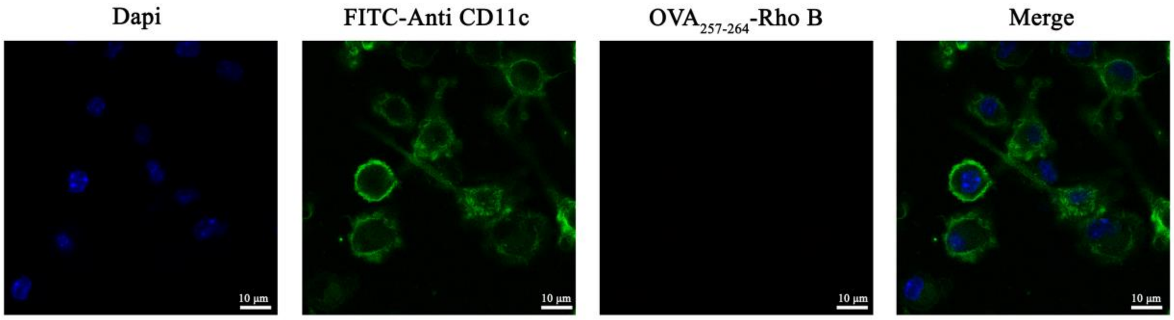

Figure S4. Cellular uptake of free $OVA_{257\text{-}264}$ by BMDCs. The cellular uptake of $OVA_{257\text{-}264}$-RhoB (red signals) by BMDCs was analyzed by CLSM measurements at 24 hours of incubation.

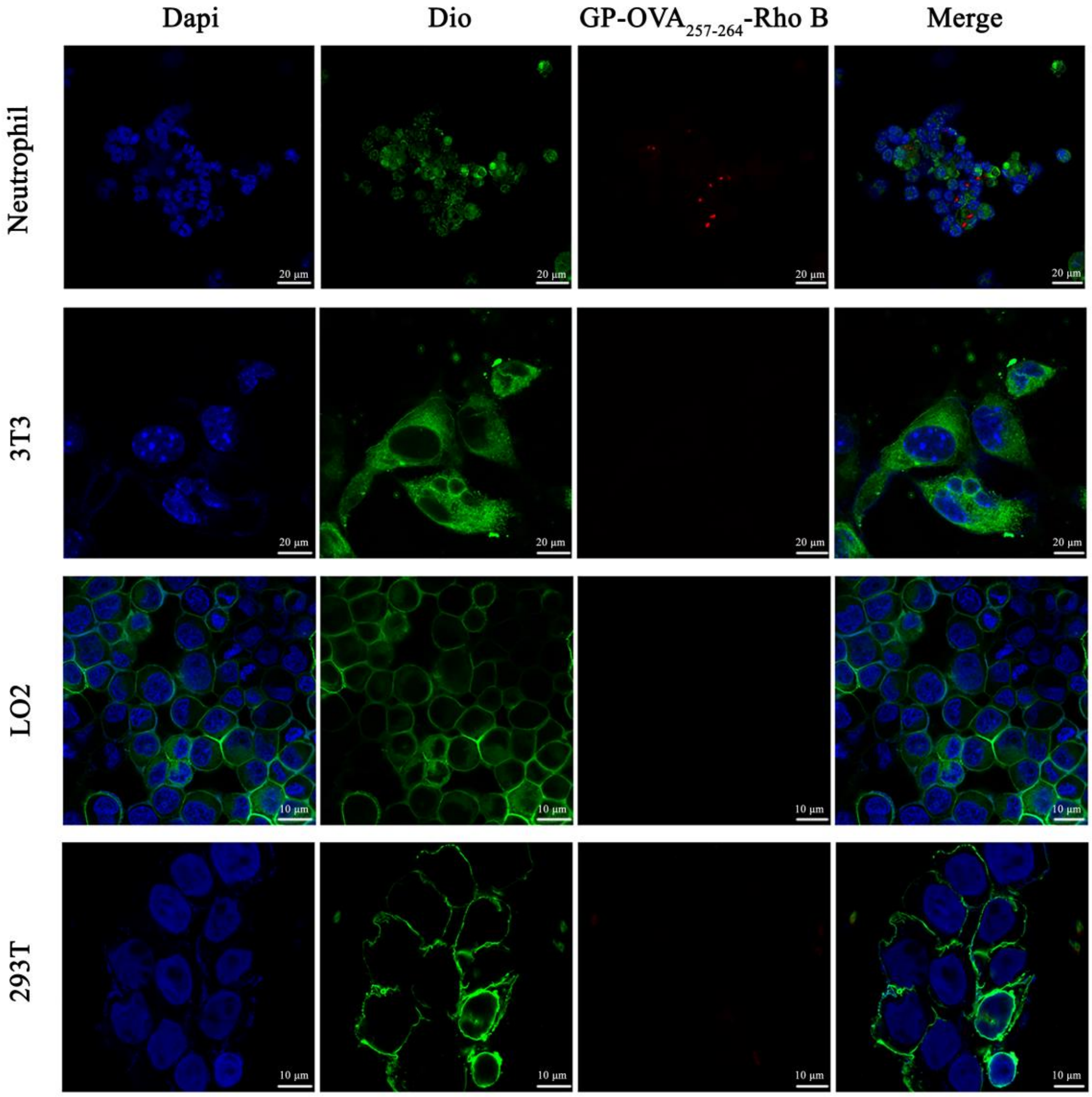

Figure S5. Cellular uptake of GP-OVA$_{257\text{-}264}$ particles by normal cells. The cellular uptake of GP-OVA$_{257\text{-}264}$-rhodamine B (red signals) by neutrophils, NIH/3T3 mouse embryo fibroblasts, LO2 human liver cells and 293T human kidney epithelial cells was analyzed by CLSM measurements at 24 hours of incubation.

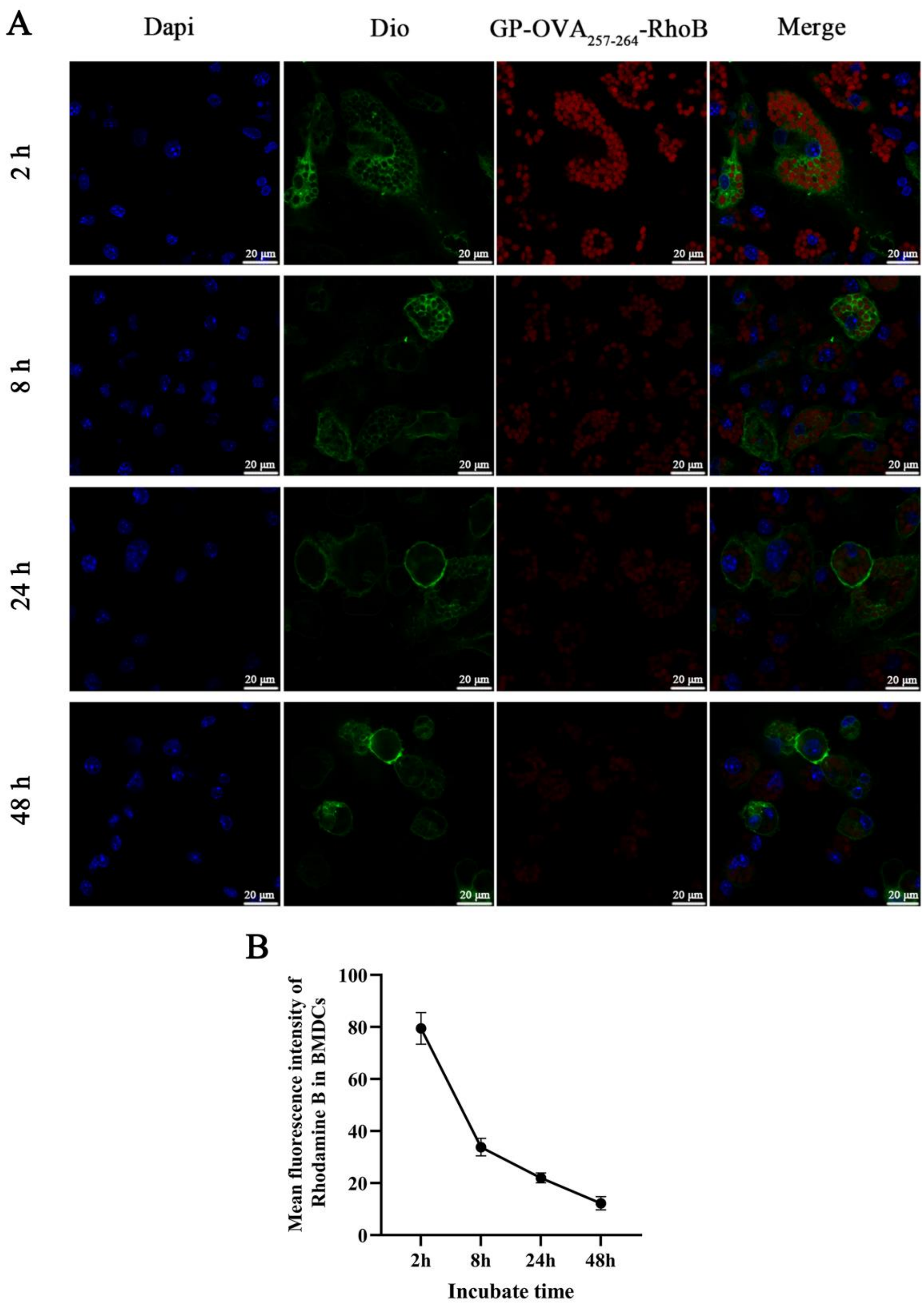

Figure S6. GP-OVA$_{257\text{-}264}$ particles were hydrolyzed by BMDCs. A) CLSM images of BMDCs after incubating with medium, OVA$_{257\text{-}264}$, GP and GP-OVA$_{257\text{-}264}$ for 2 hours, 8 hours, 24 hours and 48 hours. B) Quantified mean fluorescence intensity of RhoB in (A), n = 3.

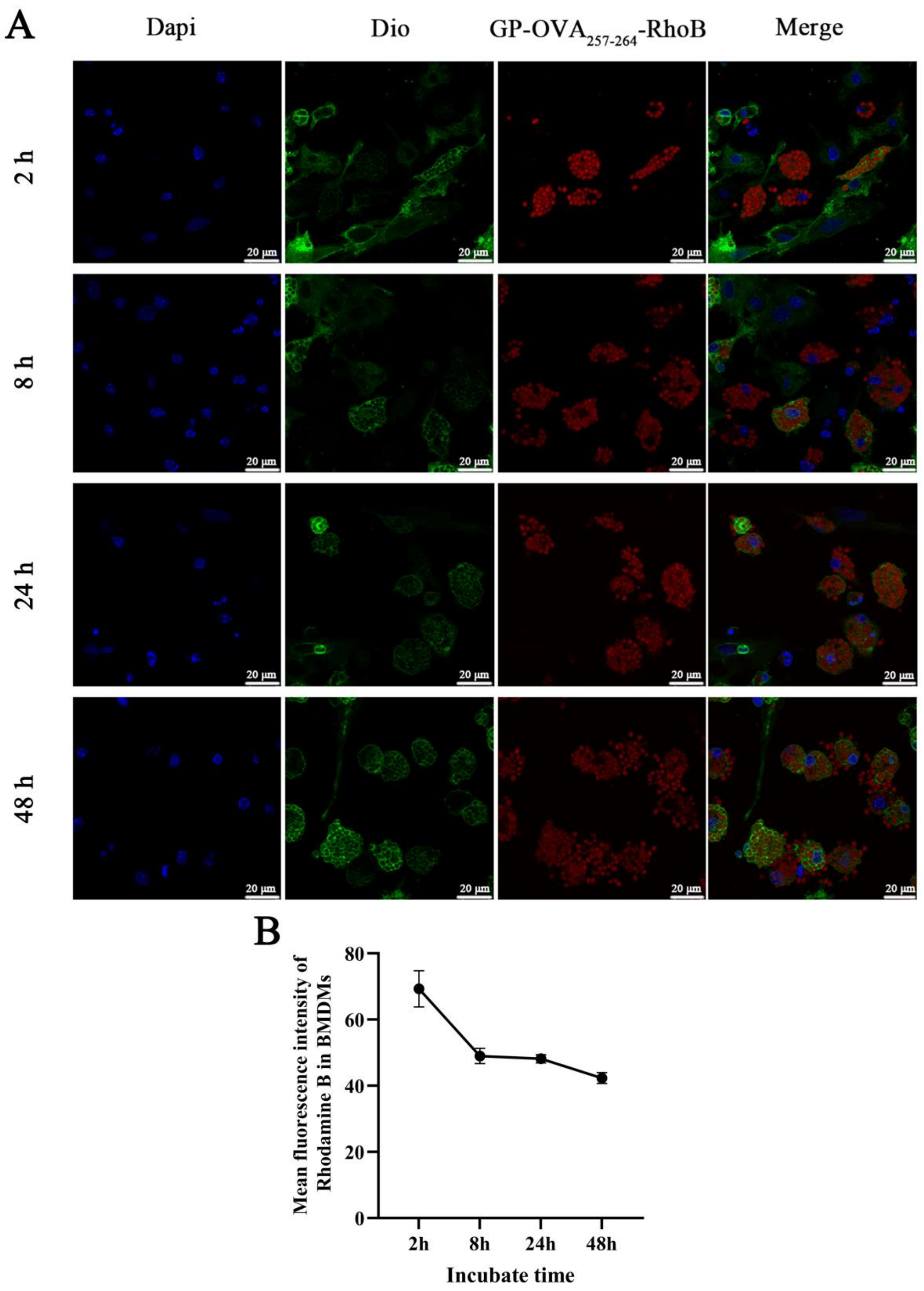

Figure S7. GP-OVA$_{257-264}$ particles were hydrolyzed by BMDMs. A) CLSM images of BMDMs after incubating with medium, OVA$_{257-264}$, GP and GP-OVA$_{257-264}$ for 2 hours, 8 hours, 24 hours and 48 hours. B) Quantified mean fluorescence intensity of RhoB in (A), n = 3.

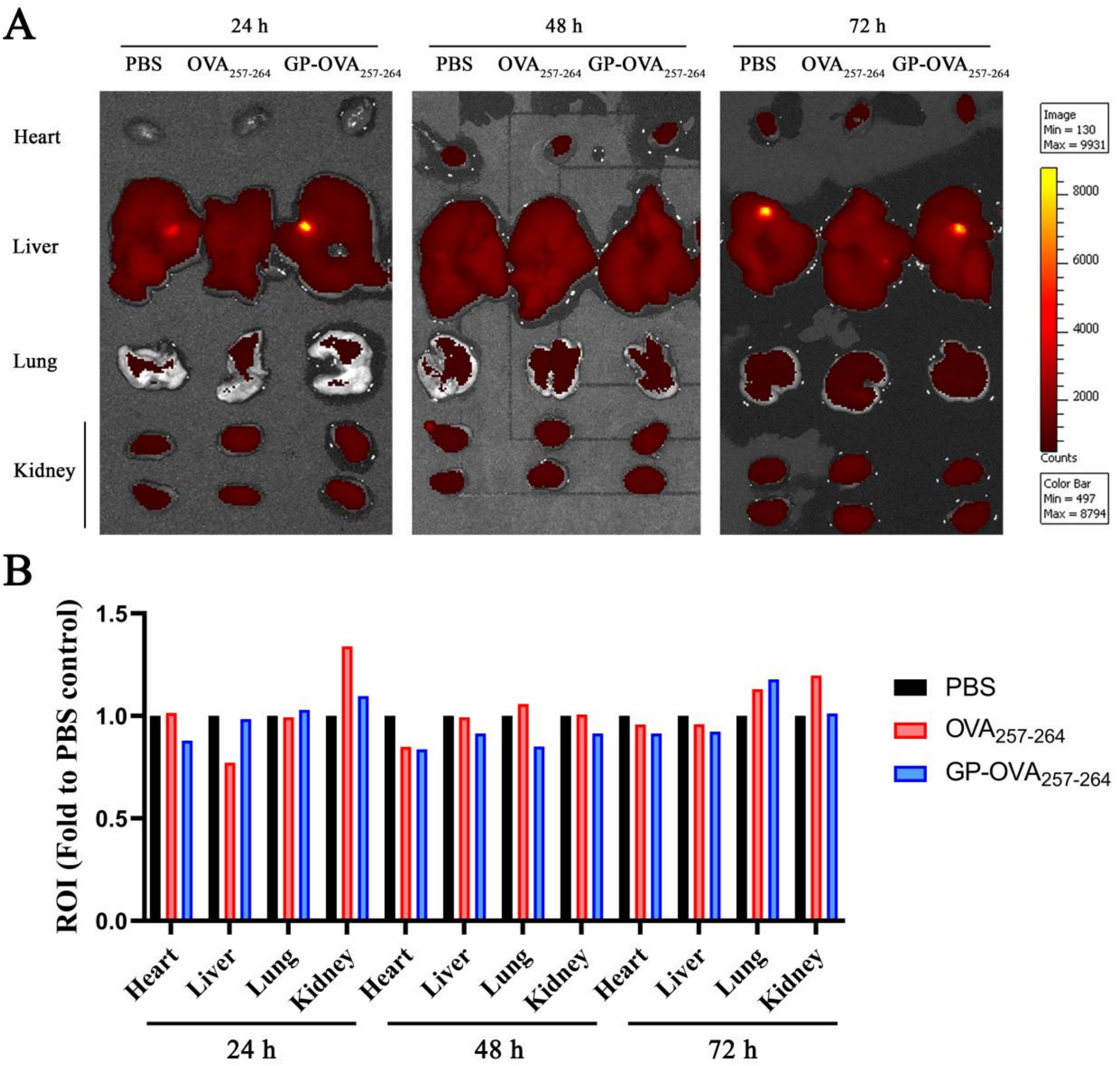

Figure S8. Uptake of GP-$OVA_{257-264}$ by normal organs in vivo. BALB/c mice were subcutaneously immunized with PBS, $OVA_{257-264}$-Cy5 or GP-$OVA_{257-264}$-Cy5. Fluorescence images of heart, liver, lung and kidney in different mice at 24 hours, 48 hours and 72 hours post-immunization are shown.

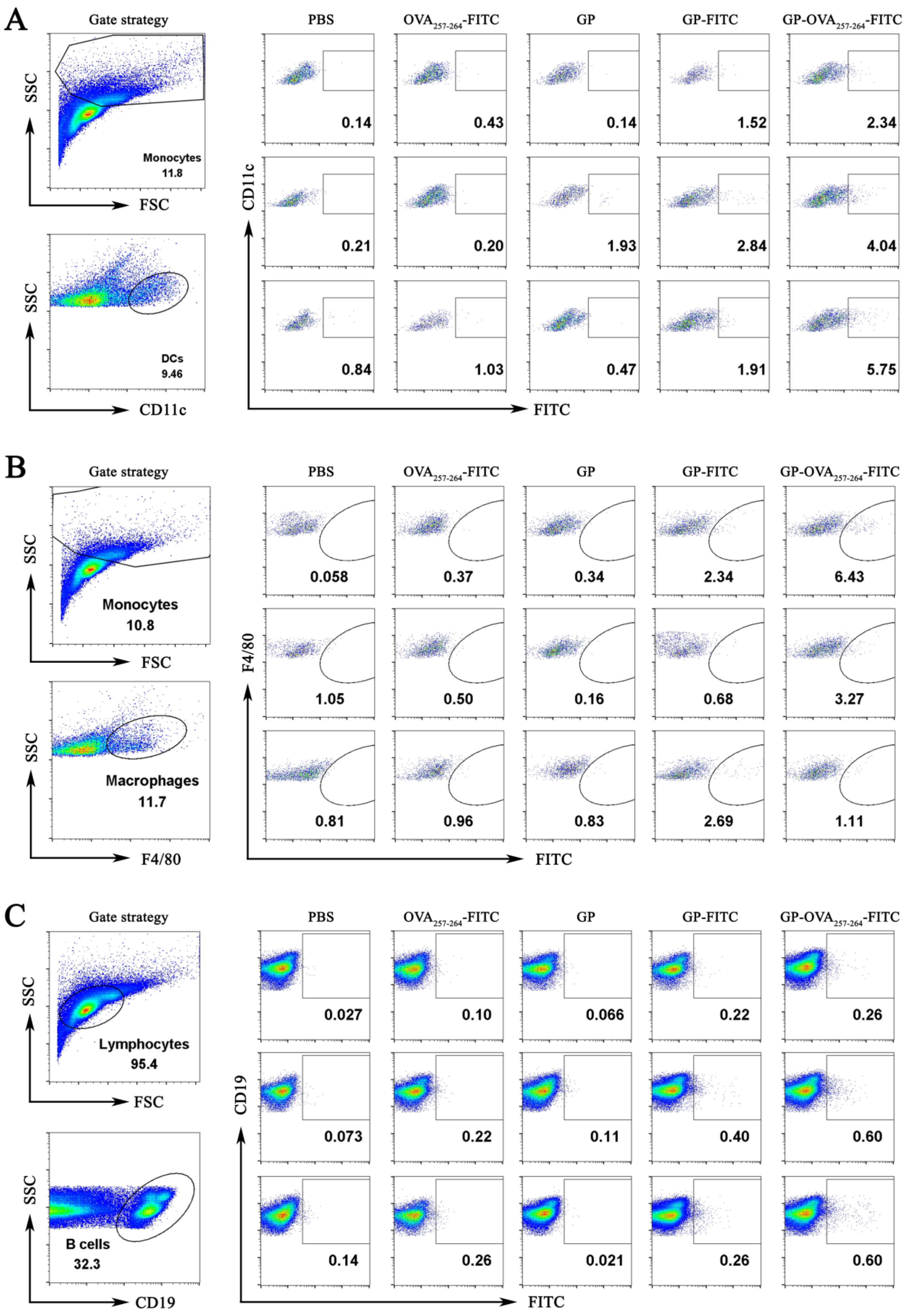
A
Gate strategy
SSC
FSC
Monocytes
11.8
DCs
9.46
CD11c
PBS
OVA257-264-FITC
GP
GP-FITC
GP-OVA257-264-FITC
0.14
0.43
0.14
1.52
2.34
0.21
0.20
1.93
2.84
4.04
0.84
1.03
0.47
1.91
5.75
FITC
B
Gate strategy
Monocytes
10.8
Macrophages
11.7
F4/80
0.058
0.37
0.34
2.34
6.43
1.05
0.50
0.16
0.68
3.27
0.81
0.96
0.83
2.69
1.11
C
Gate strategy
Lymphocytes
95.4
B cells
32.3
CD19
0.027
0.10
0.066
0.22
0.26
0.073
0.22
0.11
0.40
0.60
0.14
0.26
0.021
0.26
0.60

Figure S9. Uptake of GP-OVA$_{257-264}$ by APCs and migration to draining lymph nodes in vivo. A-C) BALB/c mice were subcutaneously immunized with PBS, OVA$_{257-264}$-FITC, GP, FITC-GP, or GP-OVA$_{257-264}$-FITC in the inguinal region. After 24 hours, the draining lymph nodes were ingested and analyzed by FACS. Flow cytometry pseudocolor graphs of dendritic cells (CD11c^{+} FITC^{+}) (A), macrophages (F4/80^{+} FITC^{+}) (B) and B cells (CD19^{+} FITC^{+}) (C) are shown.

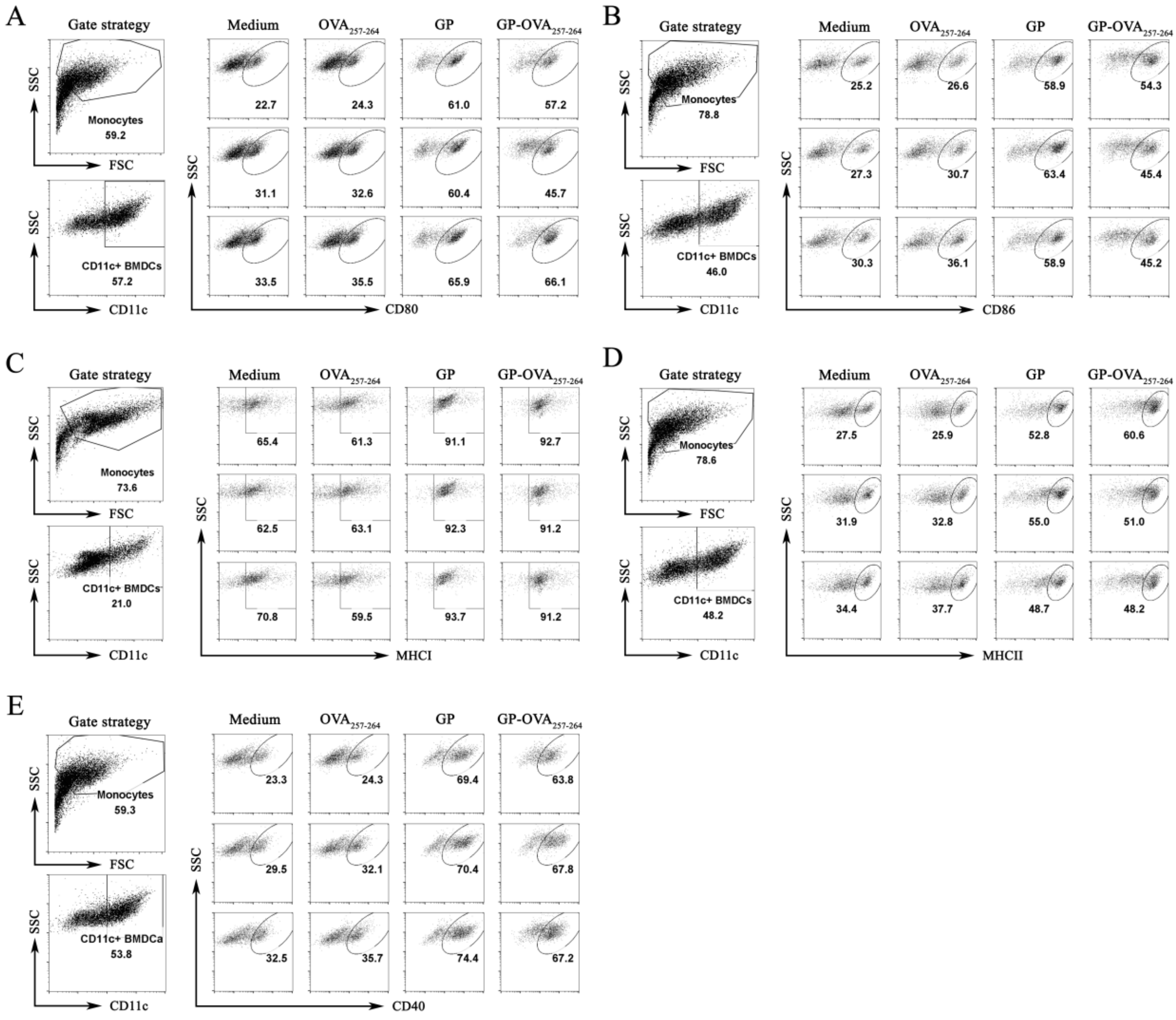

Figure S10. GP-OVA$_{257-264}$ particles induced the activation of BMDCs. A-E) FACS dot plot of CD80 (A), CD86 (B), MHC-I (C), MHC-II (D), and CD40 (E) expression in BMDCs after incubation with medium, OVA$_{257-264}$, GP and GP-OVA$_{257-264}$.

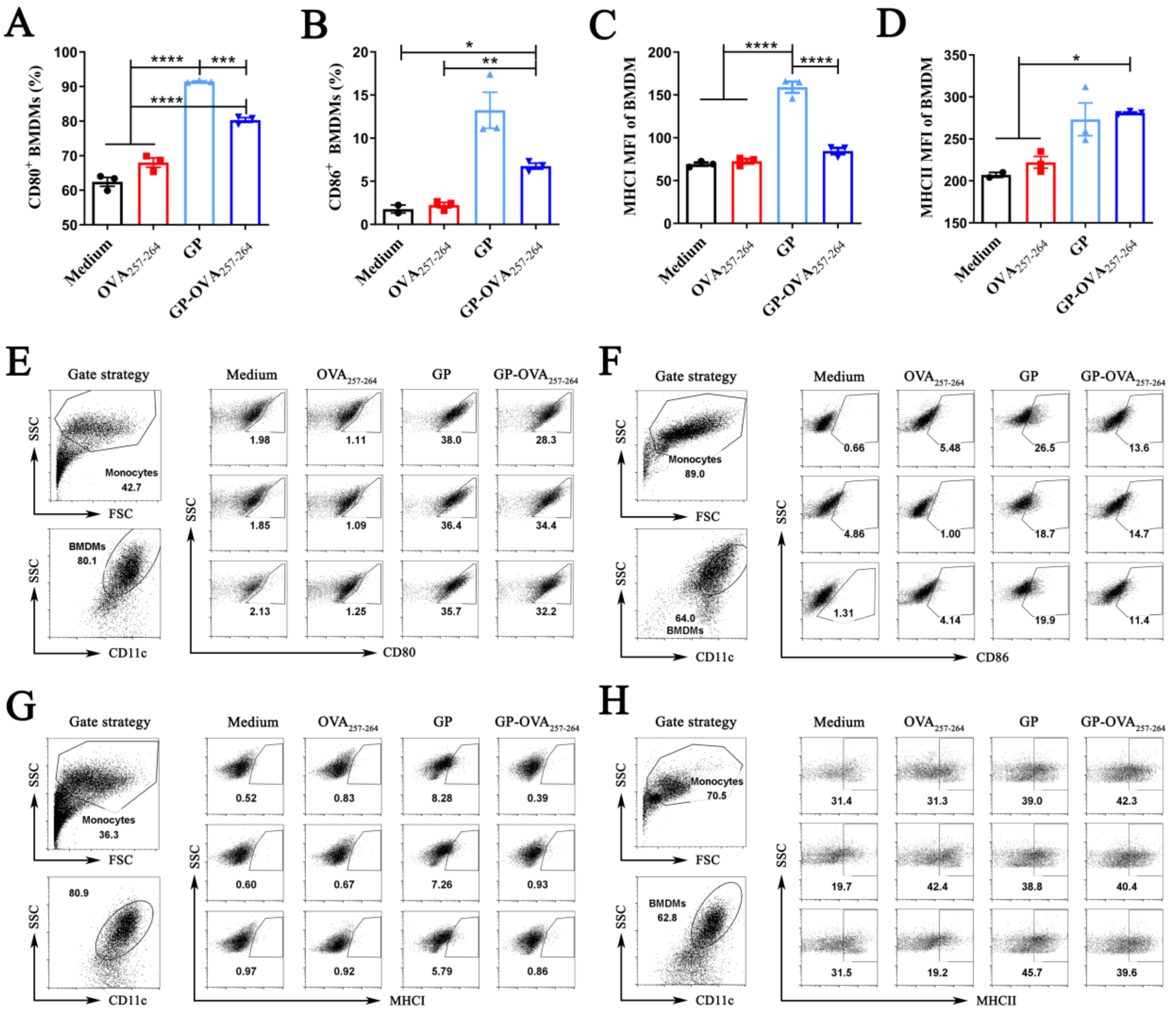

Figure S11. GP-OVA$_{257\text{-}264}$ particles induced the activation of BMDMs. A-D) Expression of the activation markers CD80 (A), CD86 (B), MHC-I (C) and MHC-II (D) in BMDMs stimulated with medium, OVA$_{257\text{-}264}$, GP or GP-OVA$_{257\text{-}264}$. E-H) Flow cytometry gate strategy and dot plot of CD80 (E), CD86 (F), MHC-I (G) and MHC-II (H) expression in BMDMs after incubation with the above vaccines. Data represent the mean ± SEM. Statistical significance was calculated by one-way ANOVA followed by a Tukey post-hoc test across groups. n = 3, * $p < 0.05$, ** $p < 0.01$, *** $p < 0.001$, **** $p < 0.0001$.

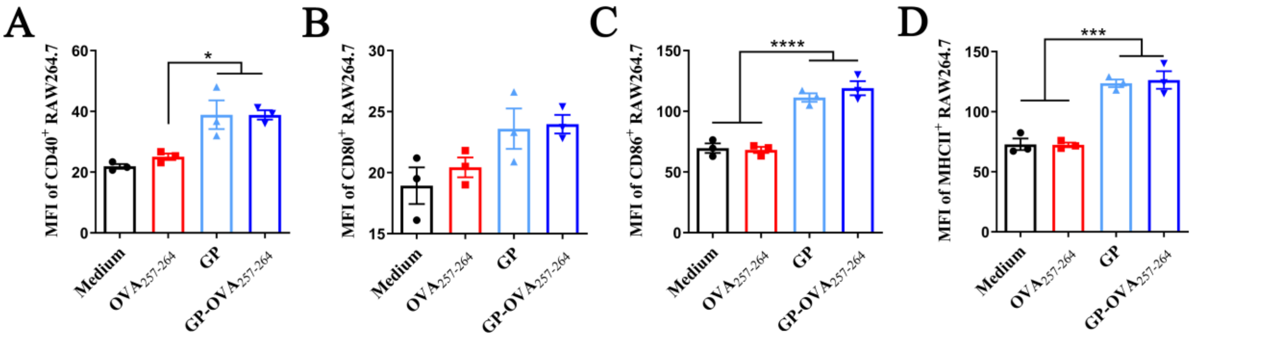

Figure S12. GP-OVA$_{257\text{-}264}$ particles induced activation of RAW264.7 cells. A-D) Mean fluorescence intensity of the activation markers CD40 (A), CD80 (B), CD86 (C) and MHC-II (D) in RAW264.7 cells stimulated with medium, OVA$_{257\text{-}264}$, GP and GP-OVA$_{257\text{-}264}$. Data represent the mean ± SEM. Statistical significance was calculated by one-way ANOVA followed by a Tukey post-hoc test across groups. n = 3, * $p < 0.05$, ** $p < 0.01$, *** $p < 0.001$, **** $p < 0.0001$.

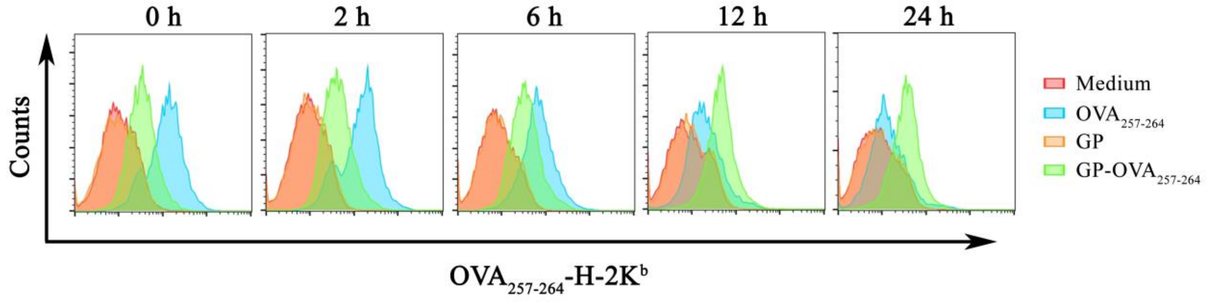

Figure S13. GP-OVA$_{257\text{-}264}$ particles induced efficient and sustainable OVA$_{257\text{-}264}$ cross-presentation. Flow cytometry histogram graphs of OVA$_{257\text{-}264}$ cross-presentation efficiency of BMDCs after incubation with medium, OVA$_{257\text{-}264}$, GP and GP-OVA$_{257\text{-}264}$ for 0 hours, 2 hours, 6 hours, 12 hours and 24 hours are shown.

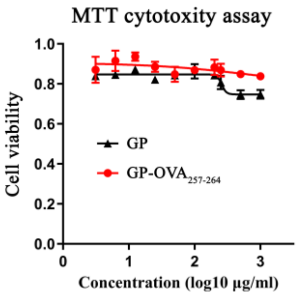

Figure S14. Cytotoxicity of GP and GP-OVA$_{257\text{-}264}$ particles to BMDCs. The viability of BMDCs incubated with different concentrations of GP and GP-OVA$_{257\text{-}264}$ was detected by MTT assay, n = 3.

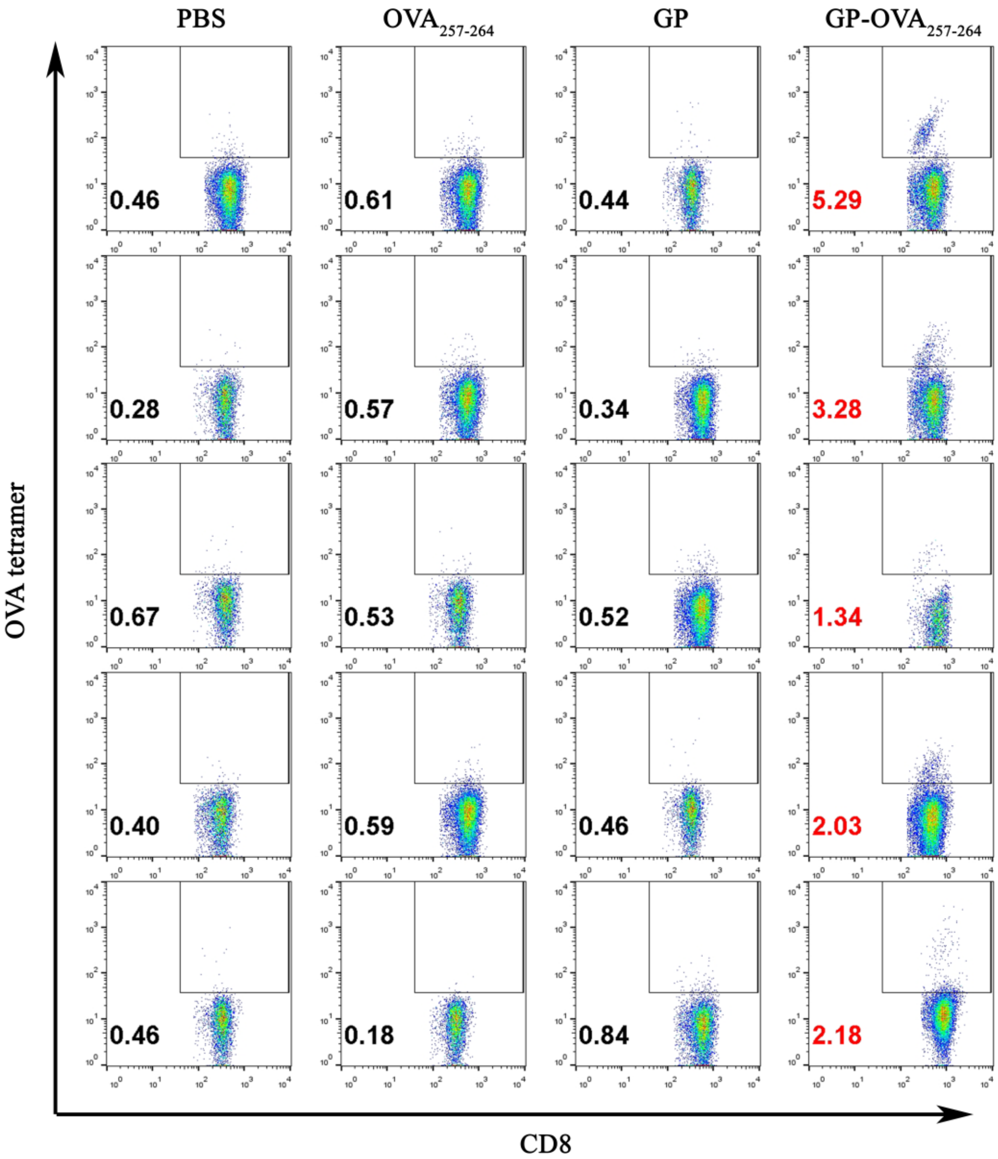

Figure S15. GP-OVA$_{257\text{-}264}$ particles induced the proliferation of OVA$_{257\text{-}264}$-specific CD8$^+$ T cells. The frequency of OVA$_{257\text{-}264}$ Tetramer$^+$ CD8$^+$ T cells in splenocytes was measured 7 days after the second immunization by OVA-tetramer staining and FACS analysis, and total pseudocolor graphs are shown, n = 5.

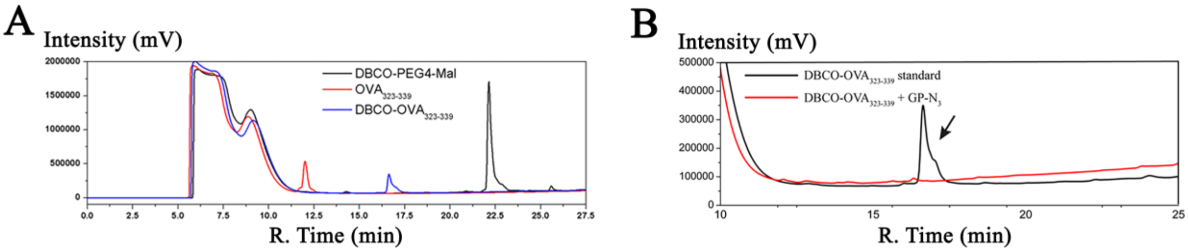

Figure S16. Monitoring of GP-OVA$_{323\text{-}339}$ preparation by HPLC. A) The reaction efficiency of cysteine-OVA$_{323\text{-}339}$ and DBCO-PEG4-maleimide was monitored. The red line indicates different standard cysteine-OVA$_{323\text{-}339}$, the black line indicates DBCO-PEG4-maleimide, and the blue line indicates the high-purity product DBCO-OVA$_{323\text{-}339}$. B) The conjugation of different DBCO-OVA$_{323\text{-}339}$ to GP-N_3 was monitored. Black and blue lines indicate standard DBCO-OVA$_{323\text{-}339}$. Arrows indicate actual DBCO-OVA$_{323\text{-}339}$. The red line indicates supernatant after the reaction.

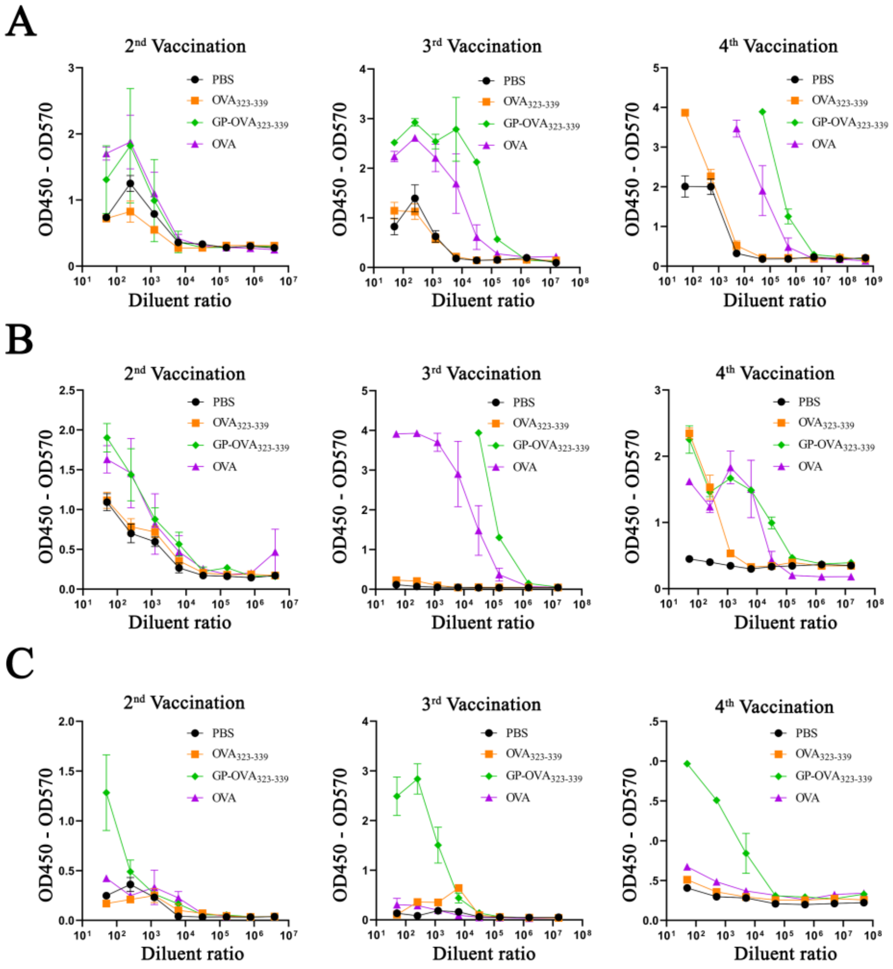

Figure S17. OVA-specific antibody titers detected by ELISA. C57BL/6 mice were inguinal immunized every two weeks with OVA$_{323\text{-}339}$, GP-OVA$_{323\text{-}339}$, OVA and PBS control. Then, 7 days after the 2nd, 3rd, and 4th immunizations, peripheral blood was obtained for ELISA

analysis of humoral immune responses. The absorbance curves of OVA-specific IgG (A), IgG1 (B) and IgG2a (C) titers are shown, n = 3.

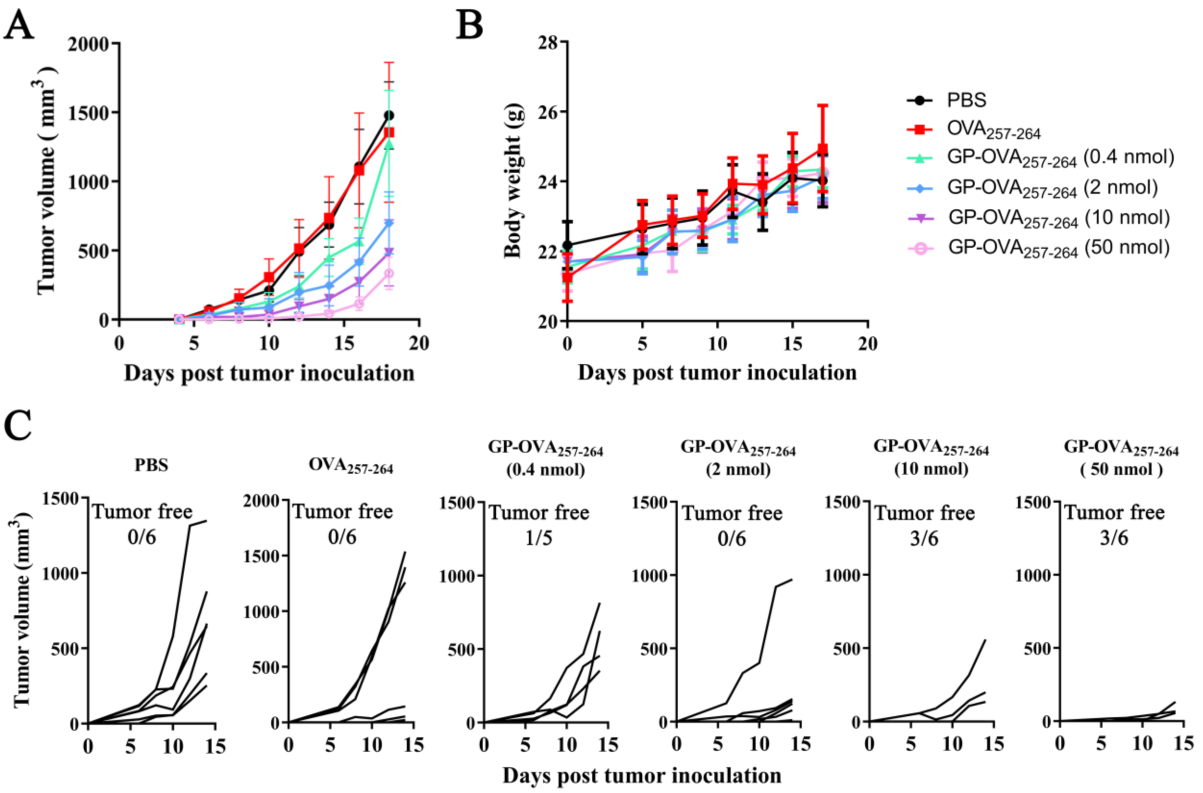

Figure S18. Equivalence ratio of $OVA_{257\text{-}264}$ to GP influences the antitumor immunity in EG7 OVA preventive tumor growth. C57BL/6 mice were subcutaneously immunized twice with gradient GP-$OVA_{257\text{-}264}$ molar weight loading efficiency (0.4 to 50 nmol $OVA_{257\text{-}264}$ / mg GPs, 5 times gradient, 4 groups) every 14 days, and PBS and 50 nmol $OVA_{257\text{-}264}$ served as controls. Seven days after the second immunization, the mice were challenged with 1 $\times 10^6$ EG7 OVA lymphoma cells. Tumor growth (A) and body weight (B) were monitored every other day, n = 6, and the tumor curve of a single mouse and proportion of tumor-free mice until Day 14 (C) are shown.

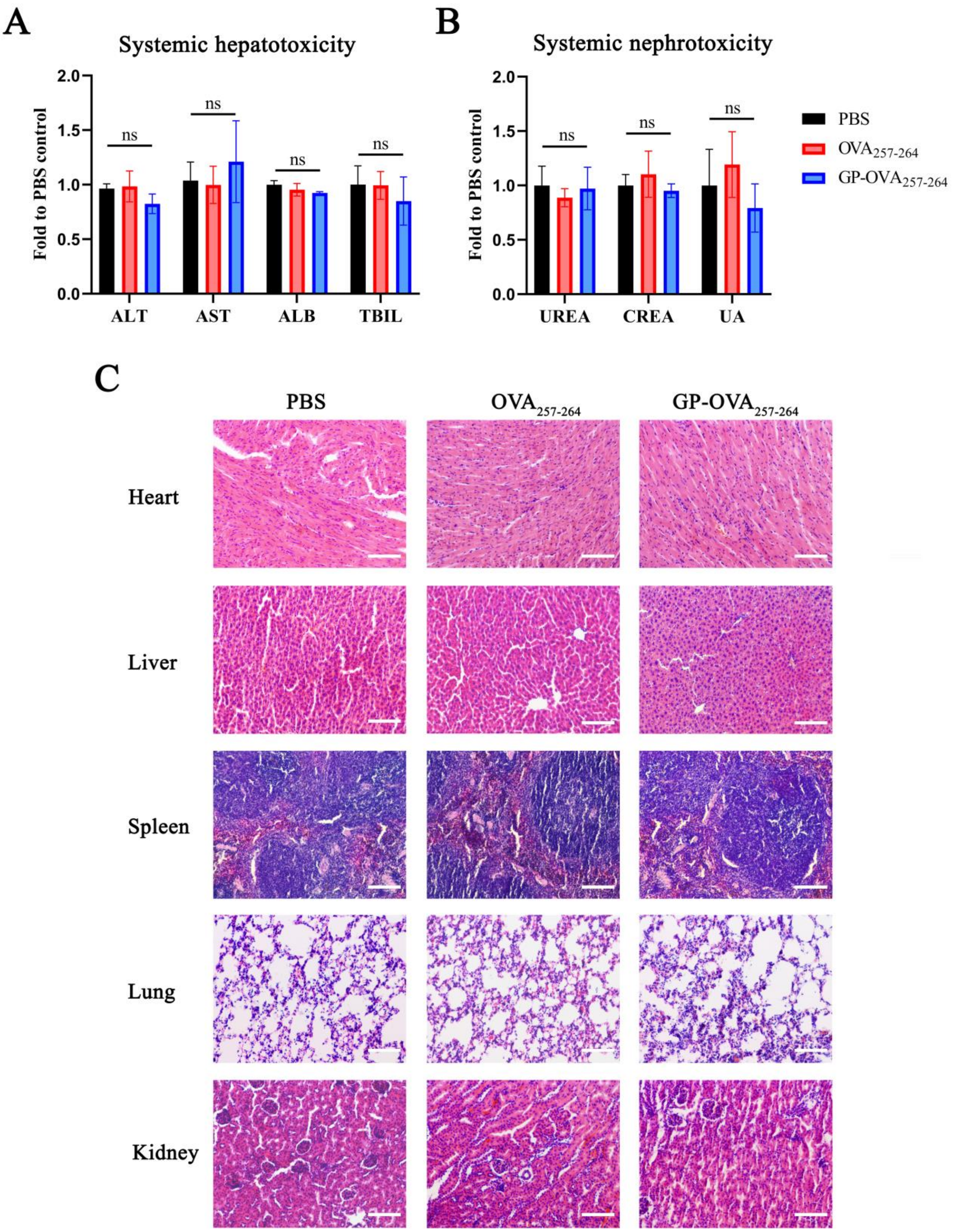

Figure S19. In vivo safety assessment of GP-OVA$_{257\text{-}264}$. C57BL/6 mice were subcutaneously immunized twice with PBS, OVA$_{257\text{-}264}$ and GP-OVA$_{257\text{-}264}$. Then peripheral blood was collected from the marginal eye vein of the mice, and A) the systemic hepatotoxicity was assessed by Chemray 240 through alanine aminotransferase (ALT), aspartate aminotransferase (AST), albumin (ALB) and total bilirubin (TBIL). B) The systemic

nephrotoxicity was assessed by Chemray 240 through UREA, creatinine (CREA) and uric acid (UA). C) H&E staining images of histopathological sections of the heart, liver, spleen, lung and kidney of immunized mice, scale bars = 100 μm. Statistical significance was calculated by one-way ANOVA followed by a Tukey post-hoc test across groups. n = 3, ns: no significant differences.

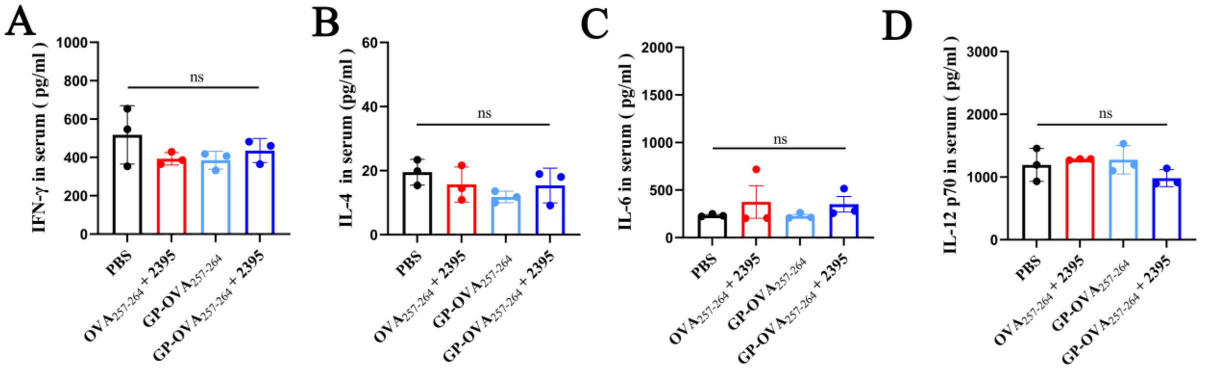

Figure S20. GP-OVA$_{257\text{-}264}$ particles minimally induced systemic cytokine secretion. C57BL/6 mice were subcutaneously immunized twice with PBS, OVA$_{257\text{-}264}$ + CpG 2395, GP-OVA$_{257\text{-}264}$ and GP-OVA$_{257\text{-}264}$ + CpG 2395. Peripheral blood was collected from the marginal eye vein of the mice, and the IFN-γ (A), IL-4 (B), IL-6 (C) and IL-12 p70 (D) levels were detected by ELISA. Statistical significance was calculated by one-way ANOVA followed by a Tukey post-hoc test across groups. n = 3, ns: no significant differences.

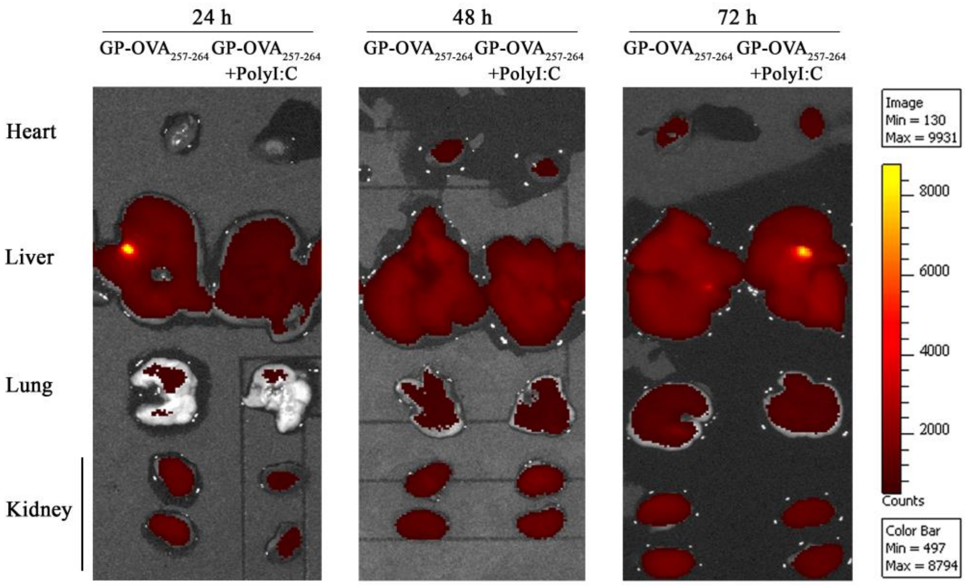

Figure S21. Uptake of GP-OVA$_{257\text{-}264}$ and PolyI:C by normal organs in vivo. BALB/c mice were subcutaneously immunized with GP-OVA$_{257\text{-}264}$-Cy5 or GP-OVA$_{257\text{-}264}$-Cy5 + PolyI:C.

Twenty-four hours, 48 hours and 72 hours after immunization, fluorescence images of the heart, liver, lung and kidney in different mice are shown.

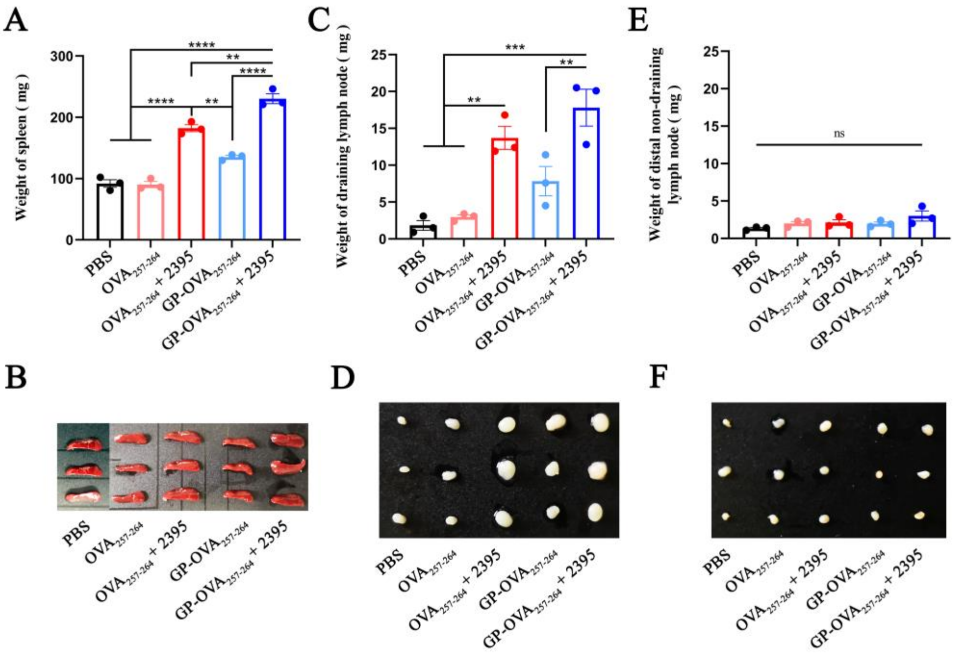

Figure S22. GP-OVA$_{257\text{-}264}$ and CpG 2395 induced swelling of the spleen and draining lymph nodes in vivo. C57BL/6 mice were immunized twice with OVA$_{257\text{-}264}$ or GP-OVA$_{257\text{-}264}$ with or without CpG 2395. Seven days after the last immunization, the mice were euthanized, and the spleens and lymph nodes were extracted. The weight (A) and comparison graph (B) of the spleen, the weight (C) and comparison graph (D) of proximal draining lymph nodes, and the weight (E) and comparison graph (F) of distal nondraining lymph nodes are shown. Statistical significance was calculated by one-way ANOVA followed by a Tukey post-hoc test across groups. n = 3 ** $p < 0.01$, *** $p < 0.001$, **** $p < 0.0001$.

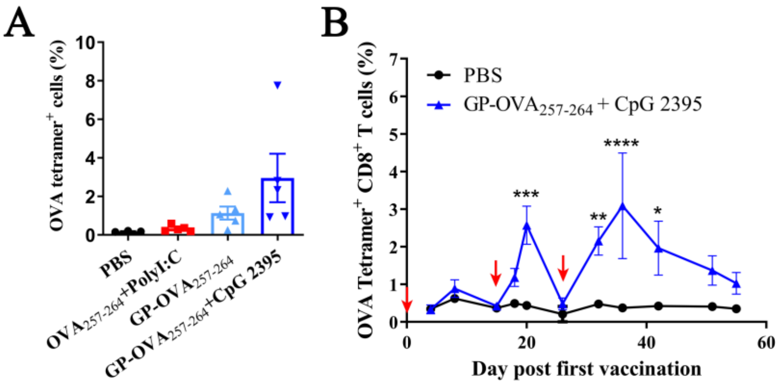

Figure S23. The combination of GP-OVA$_{257\text{-}264}$ and CpG 2395 induced stronger OVA$_{257\text{-}264}$-specific cellular immune responses. C57BL/6 mice were subcutaneously immunized twice

with PBS, $OVA_{257-264}$ + PolyI:C, GP-$OVA_{257-264}$ or GP-$OVA_{257-264}$ + CpG 2395 in the inguinal region every 14 days, and (A) the quantified frequency of $OVA_{257-264}$-specific $CD8^+$ T cells were measured by FACS 7 days after the second immunization, n = 5. (B) Then the dynamic graph of $OVA_{257-264}$-specific $CD8^+$ T cells in splenocytes was obtained based on continuous monitoring by FACS analysis. Red arrows represent the time point of immunization, n = 5. Statistical significance was calculated by one-way ANOVA followed by a Tukey post-hoc test across groups. * $p < 0.05$, ** $p < 0.01$, *** $p < 0.001$, **** $p < 0.0001$.

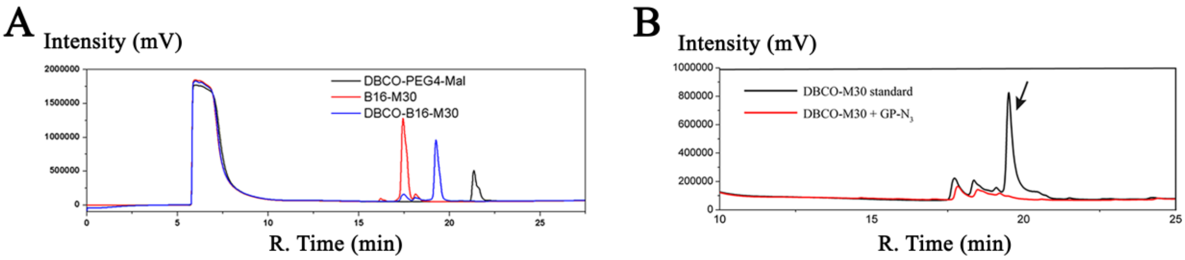

Figure S24. Monitoring of GP-M30 preparation by HPLC. A) The reaction efficiency of cysteine-M30 and DBCO-PEG4-maleimide was monitored. Red lines indicate different standard cysteine-M30, black lines indicate DBCO-PEG4-maleimide, and blue lines indicate the high-purity product DBCO-M30. B) The conjugation of DBCO-M30 to GP-N_3 was monitored. Black lines indicate standard DBCO-M30, and arrows indicate actual DBCO-M30. Red lines indicate supernatant after reaction.

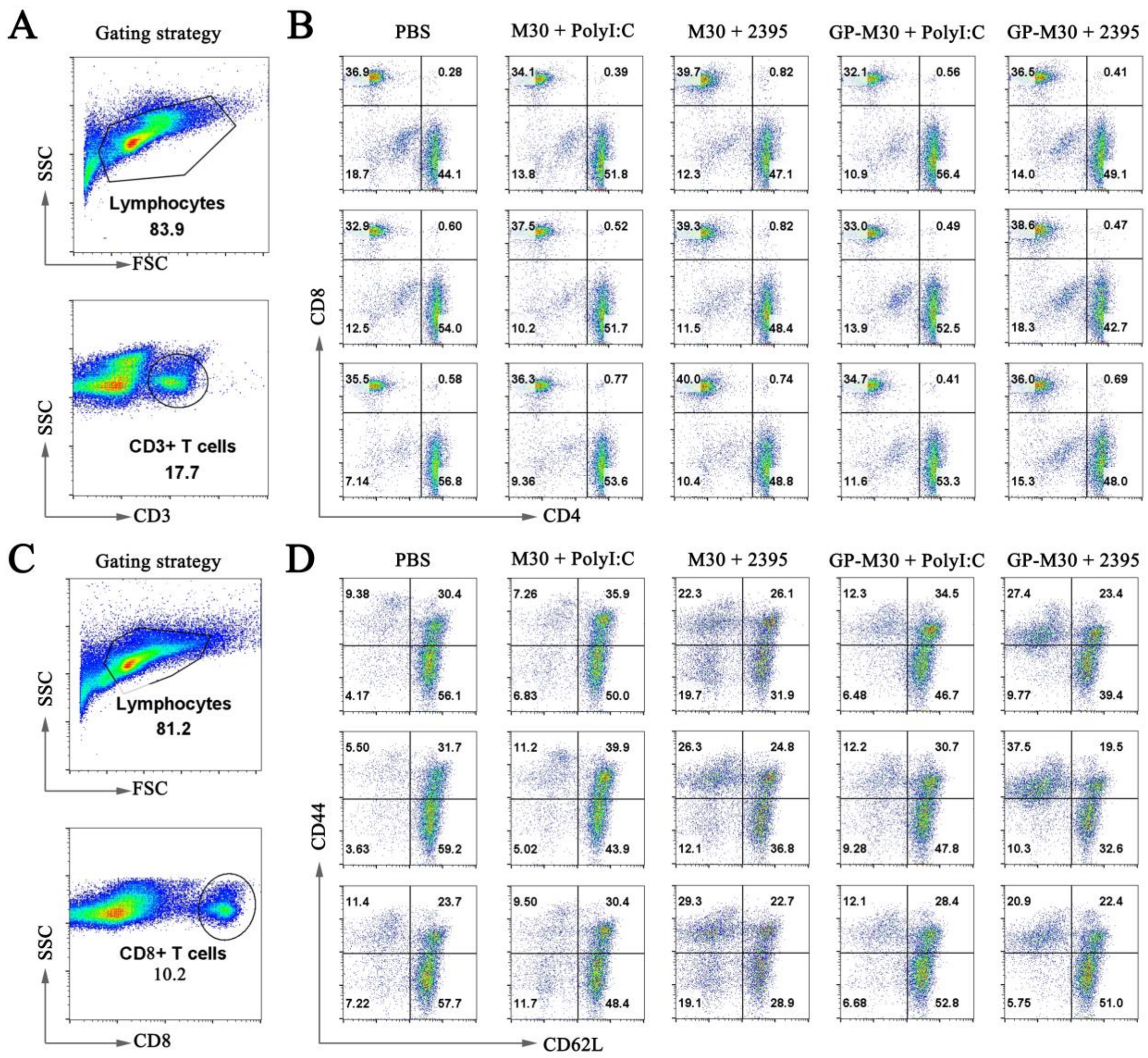

Figure S25. The combination of GP-Neoantigen with a TLR agonist induced different directions of differentiation of T cells. C57BL/6 mice were immunized twice with M30 or GP-M30 with PolyI:C or CpG 2395, and the splenocytes of immunized mice were analyzed by FACS. A, B) Gating strategy (A) and flow cytometry pseudocolor graphs (B) of $CD3^+$ T cell differentiation to $CD4^+$ T cells or $CD8^+$ T cells. C, D) Gating strategy (C) and flow cytometry pseudocolor graphs (D) of $CD8^+$ T cell differentiation into TEMs or TCMs.

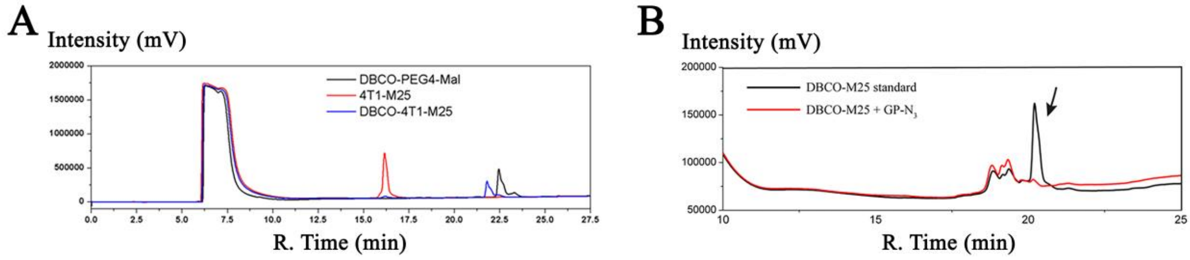

Figure S26. Monitoring of GP-M25 preparation by HPLC. A) The reaction efficiency of cysteine-M25 and DBCO-PEG4-maleimide was monitored. The red line indicates different standard cysteine-M25, the black line indicates DBCO-PEG4-maleimide, and the blue line indicates the high-purity product DBCO-M25. B) The conjugation of DBCO-M25 to GP-N_3 was monitored. The black line indicates standard DBCO-M25, and the arrows indicate actual DBCO-M25. Red line indicates supernatant after reaction.

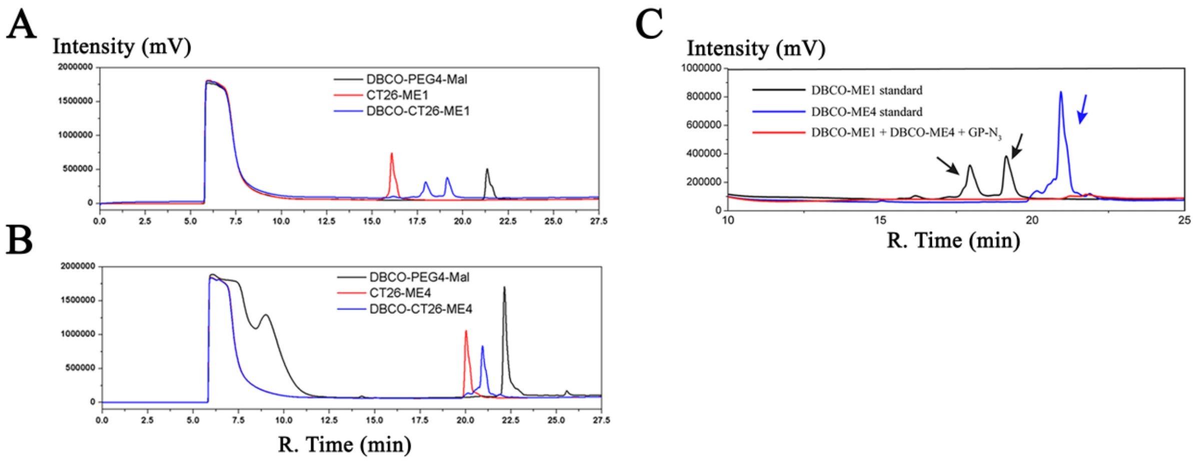

Figure S27. Monitoring of GP-ME1-ME4 preparation by HPLC. A, B) The reaction efficiency of cysteine-ME1 (A) or cysteine-ME4 (B) and DBCO-PEG4-maleimide was monitored. Red lines indicate cysteine-ME1 or cysteine-ME4, black lines indicate DBCO-PEG4-maleimide, and blue lines indicate the high-purity product DBCO-ME1 or DBCO-ME4. C) The conjugation of DBCO-ME1 and DBCO-ME4 to GP-N_3 was monitored. Black and blue lines indicate standard DBCO-ME1 and DBCO-ME4, arrows indicate actual DBCO-ME1 or DBCO-ME4, and red line indicates supernatant after reaction.
